# Integration of Care Assistants Into Intensive Care Nursing Teams: A Multimethod Study

**DOI:** 10.1155/nrp/8153123

**Published:** 2026-04-30

**Authors:** Atreyu van Esch, Dewi Stalpers, Margo M. C. van Mol

**Affiliations:** ^1^ Department of Intensive Care Medicine, Erasmus Medical Center, Rotterdam, Netherlands, erasmusmc.nl; ^2^ Department of General Practice and Nursing Science, University Medical Center Utrecht, Utrecht, Netherlands, umcutrecht.nl

## Abstract

**Aim(s):**

This study explored (1) the expectations and experiences of intensive care unit (ICU) nurses regarding the integration of care assistants, focusing on skill mix, work environment, collaboration, clinical leadership, perceived quality of care and work satisfaction, and (2) how care assistants enact and experience their role in an adult ICU environment.

**Methods:**

A multimethod, survey‐based study was conducted at two time points, using a pre–post design among 119 ICU nurses and a cross‐sectional design among 13 care assistants. The study was conducted in two adult ICUs of a large tertiary hospital. Questionnaires included self‐developed items and items derived from validated instruments (e.g., Clinical Leadership Scale, Practice Environment Scale of the Nursing Work Index and Job Content Questionnaire). Open‐ended questions were included to capture qualitative insights.

**Results:**

Response rates among ICU nurses were 79.8% (*n* = 95) at baseline and 48.7% (*n* = 58) at follow‐up, while the response rate among care assistants was 84.6% (*n* = 11). Before integration, most ICU nurses (71.6%) expected positive outcomes, such as reduced workload through the involvement of care assistants, although concerns regarding care quality were reported by 53.7%. Post‐integration, collaboration was rated positively by 82.8% of ICU nurses; however, concerns about care quality persisted (58.5%), and 55.2% opposed continuation of the initiative. Among care assistants, 54.5% found working in the ICU enjoyable, while 81.8% indicated that their assigned tasks did not align with their prior knowledge and skills. Despite these concerns, perceived quality of care was rated 8/10 by both ICU nurses and care assistants at both time points.

**Conclusion:**

Although the integration of care assistants into ICU teams was initially expected to support workforce capacity and workload distribution, persistent concerns regarding care quality and patient safety underscore the need for clearer role delineation, adequate training and structured implementation strategies to support sustainable integration.

**Summary**
 Reporting method: STROBE and COREQ
 Patient or Public Contribution: No Patient or Public Contribution

## 1. Introduction

Nurses represent the vast majority of healthcare professionals in hospitals. However, persistent nursing shortages threaten the delivery of optimal patient care, negatively affect the quality of working life and contribute to rising healthcare costs [1, 2]. Nurses also face escalating workloads potentially leading to increased absences and attrition from the profession [3–5]. This is not different in the work environment of the intensive care unit (ICU) [6]. Ensuring sufficient numbers of ICU nursing staff with high job satisfaction is essential for delivering high‐quality care and the perception of a good fit in work–life balance. To achieve this, innovative approaches are needed to reduce workload and sustain nursing care in the ICU [7]. Therefore, ICU management and nurse leaders should make efforts to understand and ameliorate the factors influencing sustainable healthcare models [8, 9].

One strategy that has gained international attention is the use of a skill mix model. Skill mix refers to a blend of grades or occupations within a team resulting in a combination of work activities or professional competencies through a variety of permitted skills [10] and could help alleviate some of the pressures faced by traditional staffing models. Such models are not new and have been applied across various healthcare settings, including critical care, by integrating registered nurses with additional caring roles such as care assistants [11–13]. The underlying rationale is that redistribution of selected care activities across different roles and responsibilities may allow ICU nurses to focus on complex patient care, while care assistants provide essential support, thus utilising maximal ICU nursing capacity.

In the Netherlands, however, the integration of care assistants within adult ICU teams has not yet been explored. Dutch care assistants are non‐registered healthcare professionals educated at European Qualifications Framework (EQF) level 3, who support nursing staff by performing delegated care activities under supervision [14]. ICU nurses are educated at EQF level 4 (vocational) or level 6 (bachelor) and retain full responsibility for patient assessment and care coordination without task division [14]. Within the ICU context, care assistants were expected to primarily support the delivery of fundamental nursing care, including personal hygiene, comfort care, positioning, nutritional support and communication with patients and families [15]. Although comparable support roles exist internationally under different titles and educational frameworks, the scope of practice, level of autonomy and degree of integration of care assistants within ICU teams vary considerably across countries and institutions.

The increasing use of skill mix models in ICUs is driven not only by nursing shortages but also by broader policy and economic pressures, including the need for workforce sustainability, cost containment and ongoing challenges in recruiting and retaining specialised ICU nurses [16, 17]. Despite these drivers and the potential efficiency gains associated with skill mix strategies, previous studies have demonstrated that working in ICU nurse–care professional dyads can be challenging for individuals from both disciplines [18, 19]. For example, time investment is needed for care assistants to sufficiently learn and provide non‐dependent basic care in the ICU environment. Successful integration, described as minimum prompts with little checking required of their work, relies on personal characteristics more than certification and predetermined task allocation. During the COVID‐19 pandemic, general nursing staff were redeployed beyond their departments to support ICU nurses in their high workload [20, 21]. ICU teams adopted the concept of ‘buddies’, comprising registered nurses with varying professional skill levels and working together in integrated teams. While these measures were deemed potentially successful by employers and policymakers, they yielded mixed positive and negative experiences for ICU nurses [20]. Therefore, establishing a skill mix in ICU nursing care requires thorough consideration of roles and the division of work tasks [22].

Evidence regarding the impact of skill mix models on patient outcomes remains mixed [23, 24]. Previous studies have shown that a higher nursing skill mix, characterised by a greater proportion of registered nurses, is associated with improved patient outcomes, including lower mortality rates and fewer nursing‐sensitive adverse events, such as delirium, pressure injuries, infections and failure to rescue [23, 24]. In contrast, Griffiths et al. found that care assistants could contribute significantly towards patient safety on general wards [25]. However, establishing the right staffing ratio is challenging and may compromise safety [25].

Clinical leadership is hypothesised to be a key challenge in the integration of new roles within the ICU; therefore, it should be taken into account during decision‐making regarding mixed‐skilled ICU teams. Defining clinical nursing leadership is an ongoing debate that includes several perspectives, styles and related concepts [26–28]. It is characterised as bedside nurses without formal authority taking a proactive role in the healthcare team to achieve positive patient outcomes or innovations in care. Clinical leadership refers to influencing good‐quality care with appropriate knowledge and competencies [29]. On the one hand, a new staffing model with a skill mix of ICU nurses’ and care assistants’ competences could allow nurses to demonstrate leadership skills and expand critical care provisions [30]. On the other hand, there could be challenges related to sharing responsibilities and recognising the competencies of unfamiliar support staff, potentially disrupting established practices and professional identities, leading to adverse mental health and intentions to leave [7, 31]. Responsibility for quality ICU care is considered as the role of the nurses, including offering fundamental care and accounting for safe protocols in the nursing domain as part of showing clinical leadership [18]. Therefore, in this study, clinical leadership has be taken into account for evaluation with regard to the integration of care assistants in the nursing team.

Welcoming additional professional disciplines requires an open attitude, optimal team work and collaborating skills among ICU nurses [32]. Enhancing a positive work environment for all ICU professionals could influence the perceived quality of care and work satisfaction regarding all care tasks performed [33, 34]. A recent review identified nine factors influencing a balanced care team, including safety, transparency and task clarity and personal relationships [35]. This provides a valuable starting point for exploring integral collaboration among care professionals, both care assistants and ICU nurses, and a new way of working among ICU nurses. Therefore, new care models, such as an integrated team with diverse skill levels, should be assessed for their impact on team collaboration, perceived quality of care and work satisfaction. While the introduction of additional caring roles may raise broader questions regarding the scope of practice, the present study focuses on experiences of collaboration and role enactment within existing professional and regulatory frameworks.

### 1.1. Aim

This study aims to explore (1) the expectations and experiences of ICU nurses with the integration of care assistants in their team, focusing on skill mix, work environment, collaboration, clinical leadership, perceived quality of care and work satisfaction and (2) how care assistants enact and experience their role in an adult intensive care environment.

## 2. Materials and Methods

### 2.1. Design

A multimethod design was adopted [36], combining quantitative survey data with embedded qualitative components derived from open‐ended questionnaire items. Data were collected using questionnaires administered at multiple time points: a pre‐post survey among ICU nurses and a cross‐sectional survey among care assistants. Although a single survey instrument was used per group, the inclusion of open‐ended questions enabled the collection of qualitative data alongside structured quantitative measures. The quantitative items identified the impact of team integration on measurable concepts, such as clinical leadership and job satisfaction, while the qualitative items explored rich, detailed insights into the personal and professional experiences of the participants [37]. This complementary design has been previously applied in ICU research to explore complex organisational and workforce interventions [38, 39]. The quantitative elements of the study were reported in accordance with the STrenghtening the Reporting of OBservational studies in Epidemiology (STROBE) [40], while qualitative findings were reported with reference to relevant consolidated criteria for reporting qualitative research (COREQ) items where applicable [41].

### 2.2. Setting and Participants

The study was conducted from August to December 2023 in a large tertiary hospital in the Netherlands, including one mixed 10‐bed medical/surgical ICU and a 16‐bed cardiothoracic ICU, both for adult patients. Participants were aged 18 or older, with at least 1 month of work experience. All participants held roles as ICU nurse or care assistant. In total, 119 certified ICU nurses and 13 care assistants were involved in the study. Individuals unable to work in the ICU during the study period, such as those on long‐term sickness or maternity leave, were excluded from participation.

### 2.3. Procedures and Data Collection

A structured approach was used to integrate care assistants into ICU teams. A steering group, consisting of nurse managers, ICU nurses and quality advisors, developed the initiative, defined care assistants’ tasks and provided structured training. All care assistants completed a 2‐week onboarding program focusing on basic ICU care, logistical support and emergency response. They were assigned to day and evening shifts under supervision of experienced ICU nurses, ensuring safe and effective collaboration.

**FIGURE 1:**
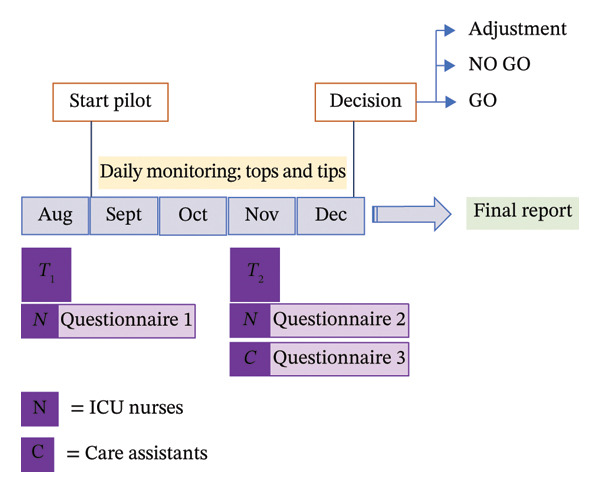
Study overview.

Data collection occurred at different time points, with specific aspects measured for each group (Figure 1). Regular team meetings facilitated feedback and adjustment throughout the study period. Questionnaires 1 and 2 were sent digitally with a 3‐month interval to all ICU nurses and nurse trainees. Questionnaire 1 was sent prior to the introduction of care assistants and took 5–10 min for completion. It primarily focused on examining expectations regarding the future integration of care assistants in the ICU. Questionnaire 2, which was sent 3 months after the introduction of care assistants, evaluated nurses’ experiences with skill mix, collaboration, quality of care, clinical leadership and work satisfaction. It took a maximum of 20 min to complete, which was allowed during work time. Questionnaire 3, which required maximal 10 min to complete, was sent to all care assistants 3 months after their start in the ICU to explore experiences with regard to skill mix, collaboration, quality of care and work satisfaction. The complete questionnaires 1, 2 and 3 are available in Supporting File 1. All questionnaires were administered using LimeSurvey and distributed by the researchers via participants’ institutional work email addresses, accompanied by a digital information sheet describing the study purpose, voluntary participation and anonymity. The email contained a request to complete the questionnaire, and informed consent was provided electronically via LimeSurvey prior to participation. No identifying information was collected, responses were not traceable to individual participants and no tracking was applied. Two general digital follow‐up reminders and QR codes provided during clinical lessons and shift handovers were used to stimulate participation. All answers were entered directly into the database.

### 2.4. Measuring Instruments

In the absence of a single validated questionnaire, which fully covered the study objectives, a combination of self‐developed items and established validated instruments was used. Self‐developed items were included to capture context‐specific aspects of integrating care assistants into ICU teams that were not adequately addressed by existing instruments. Item development was informed by available literature and the researchers’ clinical experience (AvE, MvM). Draft questionnaires were extensively discussed with the hospital’s nursing research department, refined based on feedback from ICU nurse managers and pilot tested among three ICU nurses to assess clarity, relevance and face validity.

#### 2.4.1. Expectations Integrated Teams Prior to the Introduction of Care Assistants

Questionnaire 1 included five items on demographic characteristics (gender, age, education, work experience and ICU team), four items related to work satisfaction and one item assessing perceived quality of nursing care (‘What rating would you give to the quality of nursing care on your ward?’). Expectations regarding skill mix and opinions about integrated teams including care assistants were assessed using eight self‐developed positive and negative statements. Responses were recorded on a 4‐point Likert scale (1 = ‘strongly disagree’ to 4 = ‘strongly agree’). Internal consistency of the expectation‐related items was high, with a Cronbach’s α of 0.82 (95%‐CI 0.76–0.87). Several items included free‐text fields, allowing participants to elaborate on their responses.

#### 2.4.2. Follow‐Up Measurement 3 Months After the Start of Care Assistants

Questionnaire 2 started with five items on demographic characteristics (gender, age, education, work experience and ICU team), followed by measures assessing skill mix, work environment, collaboration, clinical leadership, perceived quality of care and work satisfaction. Details on the number of items, response categories and internal consistency estimates for each construct are presented in Table 1.

**TABLE 1:** Measuring instruments including number of items, answer categories and scale reliability.

Variables	Items	Answer categories	Subscales (when applicable)	Cronbach’s α previous studies	Cronbach’s α this study (95%‐CI)
Skill mix and collaboration	11	Strongly disagree (1) – Strongly agree (4)	N/A	N/A	−0.81 (0.73–0.81)
Staffing, work environment and collaboration (PES‐NWI)	32	Strongly disagree (1) – Strongly agree (4), not applicable in my situation (5)	‐ Staffing and resource adequacy (4 items) ‐ Collegial nurse–physician relationships (7 items) ‐ Nurse manager ability, leadership and support of nurses (4 items) ‐ Nurse participation in hospital affairs (8 items) ‐ Nursing foundation of quality of care (9 items)	−0.80 −0.71 −0.84 −0.83 −0.80	−0.50 (0.26–0.68) −0.82 (0.74–0.88) −0.78 (0.67–0.86) −0.78 (0.69–0.86) −0.66 (0.51–0.78)

Perceived quality of care	1	Poor (1) – Excellent (10)	N/A	N/A	N/A
Clinical leadership scale	15	Almost never (1) – Almost always (5)	‐ Overall ‐ Challenging the process (3 items) ‐ Inspiring a shared vision (3 items) ‐ Enabling others to act (3 items) ‐ Modelling the way (3 items) ‐ Encouraging the heart (3 items)	−0.86 −0.64 −0.70 −0.72 −0.66 −0.78	−0.85 (0.78–0.90) −0.44 (0.14–0.65) −0.66 (0.48–0.79) −0.59 (0.37–0.75) −0.44 (0.14–0.65) −0.92 (0.87–0.95)

Clinical leadership – Self perceived	2	Agree – Disagree	N/A	N/A	N/A
Work satisfaction – Job content questionnaire	3	Very dissatisfied (1) – Very satisfied (4)	N/A	N/A	N/A
Intention to leave – Job content questionnaire	3	Yes – No	N/A	N/A	N/A

*Note:* N/A = not applicable/available; PES‐NWI = Practice Environment Scale of the Nursing Work Index.

##### 2.4.2.1. Skill Mix

ICU nurses’ experiences with integrated teams and perceived skill mix were assessed using 11 self‐developed statements. These statements were supplemented with two open‐ended questions to explore experiences on skill mix and one item assessing nurses’ willingness to continue the initiative.

##### 2.4.2.2. Work Environment and Collaboration

An appropriate and psychometrically robust instrument to measure work environment is still inconclusive [42, 43]. In the current study, work environment was measured using the Dutch version of the 32‐item validated Practice Environment Scale of the Nursing Work Index (PES‐NWI) [44, 45]. The items were categorised into five subscales representing different aspects of the working environment.

##### 2.4.2.3. Clinical Leadership

Clinical leadership was measured with the validated Dutch version of the Clinical Leadership Scale (CLS) [46, 47]. The CLS consists of 15 items divided into 5 subscales: challenging the process, inspiring a shared vision, enabling others to act, modelling the way and encouraging the heart. In addition, two self‐perceived clinical leadership items were included, allowing respondents to indicate agreement or disagreement.

##### 2.4.2.4. Perceived Quality of Care

Perceived quality of care was assessed using a single‐item measure, responses ranging from 1 (poor quality of care) to 10 (excellent quality of care).

##### 2.4.2.5. Work Satisfaction

Work satisfaction was assessed using three items derived from the Job Content Questionnaire, followed by three items assessing intention to leave and two items evaluating the likelihood of recommending the hospital as a workplace to ICU nurse colleagues and as a care facility to families [48, 49].

#### 2.4.3. Follow‐Up Measurement for Care Assistants 3 Months After Starting at the ICUs

Questionnaire 3 included four demographic items (gender, age, work experience and ICU team), nineteen self‐developed statements regarding experiences of working in an ICU, four items assessing work satisfaction and one item measuring perceived quality of care. Responses were recorded on a 4‐point Likert scale (1 = ‘strongly disagree’ to 4 = ‘strongly agree’), with opportunities for additional free‐text comments. The internal consistency of the 19‐item experience scale demonstrated acceptable reliability, with a Cronbach’s α of 0.77 (95%‐CI: 0.51–0.93).

### 2.5. Analysis

#### 2.5.1. Quantitative

No a priori sample size calculation was performed, as data were collected from the entire ICU nursing team. Data were analysed using descriptive statistics in R Project for Statistical Computing (version 2023.06.0), based on original data, and, when applicable, according to established cut‐offs or scale labels. Descriptive statistics (absolute numbers and proportions) were used to summarise demographic characteristics and to present noticeable differences between groups. To assess demographic differences between the two time points, categorical variables (e.g. gender and the ICU team) were analysed using Chi‐square tests, while ordinal variables (e.g. age and work experience) were compared using Mann–Whitney *U* tests. All tests were two‐tailed, and a *p* value < 0.05 was considered statistically significant. To evaluate (sub) scale reliability of the several constructs, Cronbach’s alpha was calculated for each subscale separately, assuming unidimensionality. For the PES‐NWI and the CLS, mean scores and standard deviations (SDs) were computed per scale and subscale. Subscales and overall score (PES‐NWI total) are presented as a composite measure, calculated by averaging the items (scores ranging from 1 to 4). A cut‐off value of ≥ 2.5 indicates a positive assessment of the work environment [44, 45]. For inclusion in the PES‐NWI subscales, at least half plus one of the items ((n/2) + 1) needed to be completed. As a result, only one respondent was excluded from the *Nurse manager ability* subscale, while two respondents were excluded from the *Nurse participation in hospital affairs* subscale. Responses marked as ‘not applicable’ were treated as missing values. Missing data were imputed using the population mean for the respective item. The total CLS score ranged from 15 to 75, with higher scores indicating stronger leadership behaviours, and was calculated as the sum of all 15 items per participant [46, 47]. For each subscale, a composite score was computed by averaging the corresponding three items, yielding a score between 1 and 5. A cut‐off value of ≥ 3.0 was used to indicate a positive assessment of clinical leadership skills and behaviours. All participants completed the CLS items, resulting in no missing data.

#### 2.5.2. Qualitative

Due to the short time frame for evaluation of the intervention, a rapid analysis of data was undertaken [50], as qualitative data collection and analysis were conducted in parallel. This method offered flexibility to explore and re‐evaluate participants’ insights during ongoing data collection. In addition, the rapid analysis enabled iterative data validation while conducting the study between both timepoints [51]. The data were analysed using a deductive approach in AtlasTi, including minimally describing the data set in rich detail and representing some level of patterned response or meaning within the data set [52]. To ensure rigour, the entire process of analysis was performed by two independent researchers (AvE and MvM, both familiar with the ICU nursing practice).

### 2.6. Ethical Considerations

Ethical approval for this study was waived by the Medical Ethics Committee (MEC‐2023‐0551), who determined that the specifications outlined in the Dutch Medical Research Involving Human Subjects Act were not applicable. Both oral and written informed consent were obtained from all participants. They were informed of their right to withdraw from the study at any time. The collected data were processed in a pseudo anonymised manner to ensure participants’ privacy.

## 3. Results

### 3.1. Demographic Characteristics

A total of 95 ICU nurses completed questionnaire 1 (response rate 79.8%), of which 68 (71.6%) were women and most were aged between 20 and 30 years (*n* = 25, 26.3%). A majority hold a basic EQF level 4 qualification extended with an ICU specialisation (*n* = 39, 41.1%), while the most common work experience was between 1 and 5 years and over 21 years (both *n* = 27, 28.4%). A total of 58 ICU nurses completed questionnaire 2 (response rate 48.7%). Work experience differed between the two time points (*p* = 0.006), with a decrease in ICU nurses with less than 1 year of experience (20.0% before vs. 3.4% after start of the care assistants) and an increase in those with 11–20 years of experience (11.6% and 29.3%, respectively). No significant differences were observed for gender, age, education and the ICU team. Questionnaire 3 was completed by 11 out of 13 care assistants (response rate 84.6%). All demographic characteristics are outlined in Table 2.

**TABLE 2:** Demographic characteristics of the participants.

Variables	ICU nurses			Care assistants
	Baseline *T* = 1 (*N* = 95) (%)		Follow‐up *T* = 2 (*N* = 58) (%)	(*N* = 11) (%)
Gender, female	68 (71.6)		41 (70.7)	9 (81.8)

*Age*				
20–30 years	25 (26.3)		12 (20.7)	4 (36.4)
31–40 years	21 (22.1)		16 (27.6)	2 (18.2)
41–50 years	9 (9.5)		11 (19.0)	3 (27.3)
51–60 years	18 (18.9)		15 (25.9)	2 (18.2)
Older than 60 years	11 (11.6)		4 (6.9)	—
Prefer not to say	2 (2.1)		—	—

*Education*				
Nurse EQF level 4 with ICU specialisation	45 (47.4)		28 (48.3)	—
Nurse EQF level 6 in with ICU specialisation	49 (51.6)		30 (51.7)	
Unknown	1 (1.1)		—	

*Work experience – ICU nurses* ^∗^				
Less than 1 year	19 (20.0)		2 (3.4)	—
1–5 years	27 (28.4)		13 (22.4)	
6–10 years	11 (11.6)		6 (10.3)	
11–20 years	11 (11.6)		17 (29.3)	
More than 21 years	27 (28.4)		20 (34.5)	

*Work experience – care assistants*				
Less than 1 month		—		1 (9.1)
1–2 months				1 (9.1)
2–3 months				6 (54.5)
More than 3 months				3 (27.3)

*ICU team*				
Medical/surgical adult ICU		24 (25.3)	19 (32.8)	7 (63.6)
Cardiothoracic adult ICU		71 (74.7)	39 (67.2)	4 (36.4)

*Note:* Differences were tested with a Chi‐squared test or Mann–Whitney U test, categorical variables are presented as frequency (%); T = Time point.

Abbreviations: EQF = European Qualification Framework, ICU = intensive care unit.

^∗^ *p* < 0.05.

### 3.2. Expectations Regarding Integrated Teams

**FIGURE 2:**
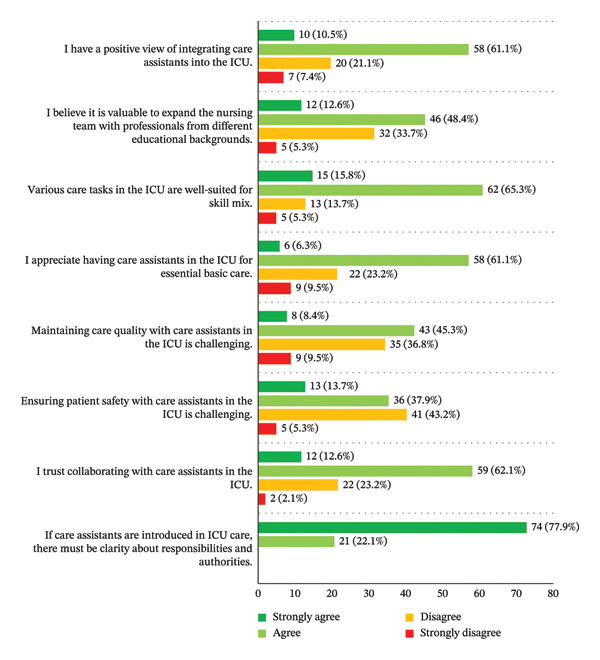
ICU nurses’ expectations on skill mix and opinions about integrated teams.

The majority (*n* = 68, 71.6%) was positive about the involvement of care assistants within the team, and 81.1% (*n* = 77) reported that various ICU‐related tasks were suitable for a skill mix. Perceived quality of care was 8 [IQR 7–8]. However, 53.7% of participants (*n* = 51) expressed concerns about maintaining the quality of care, and 51.6% (*n* = 49) expected that ensuring patient safety might be compromised. Almost all reported that clarity on responsibilities and authorities is fundamental when integrating care assistants into ICU care (*n* = 74, 77.9% agreed; *n* = 21, 22.1% strongly agreed) (Figure 2).

#### 3.2.1. Narratives of Expectations to Integrated Teams

ICU nurses expressed optimism and highlighted that care assistants could help with routine tasks, allowing ICU nurses to focus on more specialised care. For example, one respondent noted that ‘*It’s nice to have extra hands to take care of basic tasks, which gives us more time to focus on our specialisation’.* There was also recognition that care assistants could help reduce the workload during staff shortages: ‘*In the future, when ICU nurse shortages arise, it will be helpful to have colleagues who can at least manage basic care’.*

Despite this optimism, ICU nurses also expressed caution, emphasising that the success of this intervention would depend on adequate training and clear role delineation. As one respondent noted, ‘*It will be challenging for both ICU staff and care assistants to find their footing at first, but with clear responsibilities, this could work well’*. Concerns were particularly raised regarding patient safety and clinical decision‐making, especially in acute situations. One participant stated, ‘*I worry about moments when care assistants may not know when to involve an ICU nurse, especially in urgent situations requiring immediate action’.* (See Supporting File 2 for additional comments)

### 3.3. Participants’ Experiences Addressing to Work in Integrated Teams

**FIGURE 3:**
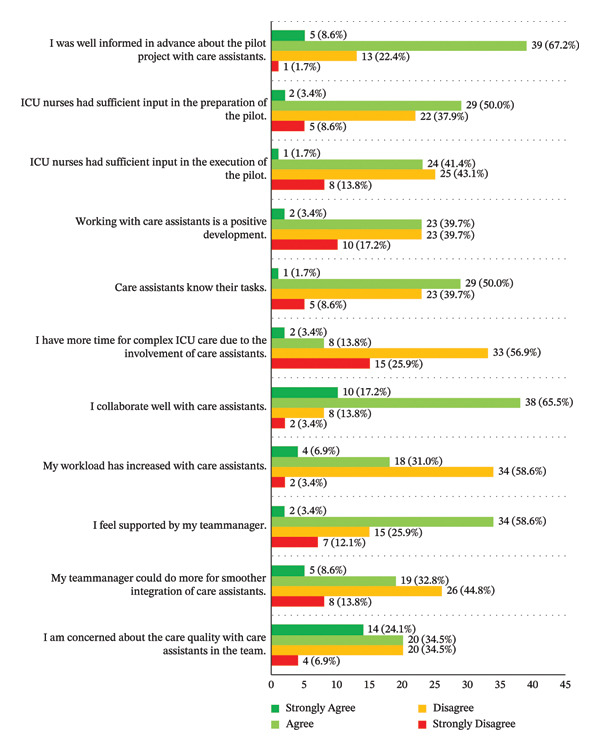
ICU nurses’ evaluation of integrated teams with care assistants.

A majority of ICU nurses (*n* = 44, 75.9%) reported being well‐informed in advance about the work in integrated teams. While 62.1% (*n* = 36) did not experience an increase in workload following the integration of care assistants, 82.8% (*n* = 48) reported that they did not gain additional time to provide complex ICU care (Figure 3). Whether the integration of care assistants in the ICU should be established as a permanent structure, 44.8% of participants (*n* = 26) were in favour, while 55.2% (*n* = 32) opposed the continuation.

Care assistants predominantly showed positive experiences regarding their work in the ICU. Most care assistants (*n* = 10, 90.9%) found the information they received about working in the ICU to be sufficient to good. In addition, 63.6% (*n* = 7) reported feeling welcomed. A majority (*n* = 6, 54.5%) experienced working in the ICU as an enjoyable challenge, with two (18.2%) finding it too stressful. Regarding the alignment between the work in the ICU and their knowledge and skills, a majority (*n* = 9, 82%) indicated that the work did not match sufficiently (Table 3).

**TABLE 3:** Experiences of care assistants working in the ICU.

Statements	Strongly disagree (%)	Disagree (%)	Agree (%)	Strongly agree (%)
The information I received about working in the ICU was sufficient for me.	—	1 (9.1)	9 (81.8)	1 (9.1)
I felt welcomed in the ICU.	1 (9.1)	3 (27.3)	6 (54.5)	1 (9.1)
I was properly trained in ICU care.	—	3 (27.3)	6 (54.5)	2 (18.2)
I know which tasks I need to perform.	—	2 (18.2)	8 (72.3)	1 (9.1)
I feel comfortable performing the tasks assigned to me.	—	—	11 (100)	—
Collaboration with ICU nurses is generally pleasant.	—	1 (9.1)	9 (81.8)	1 (9.1)
I find working in an ICU usually a fun challenge.	—	—	5 (45.4)	6 (54.5)
I find working in an ICU (too) stressful.	4 (36.4)	5 (45.5)	2 (18.2)	—
I feel that I am overly directed by ICU nurses in the tasks I perform.	2 (18.2)	9 (81.8)	—	—
I feel uncertain in collaborating with ICU nurses.	2 (18.2)	4 (36.4)	5 (45.5)	—
I know when to alert an ICU nurse.	1 (9.1)	1 (9.1)	7 (63.6)	2 (18.2)
The work in the ICU aligns with my knowledge and skills.	—	9 (81.8)	2 (18.2)	—
I find it easy to support families in the ICU, such as offering a diary or a comforting conversation.	1 (9.1)	4 (36.4)	6 (54.5)	—
I would recommend working in an ICU to fellow care assistants.	—	3 (27.3)	6 (54.5)	2 (18.2)
I feel valued as a colleague in the ICU team.	—	2 (18.2)	8 (72.7)	1 (9.1)
I experience high work pressure.	7 (63.6)	4 (36.4)	—	—
I feel supported by my supervisor.	—	—	8 (72.7)	3 (27.3)
My supervisor could do more to facilitate smoother team integration.	—	7 (63.6)	3 (27.3)	1 (9.1)
I feel like I am part of the care for ICU patients/I contribute (professionally) to the care of ICU patients.	—	4 (36.4)	7 (63.6)	—

Abbreviation: ICU = intensive care unit.

#### 3.3.1. Skill Mix and Nursing Work Environment

Although 48.3% of ICU nurses (*n* = 28) (strongly) agreed that support services in general allowed them to spend adequate time with patients, 46.6% (*n* = 27) felt that these services did not increase the time available for direct patient care. In addition, 94.8% (*n* = 55) reported working with clinically competent nurses, and 81.0% (*n* = 47) reported that there were enough registered nurses to provide quality patient care. The overall PES‐NWI score had a mean of 2.89 [SD = 0.31], indicating a positive assessment of the work environment. Among the five subscales, the highest mean score was found for the subscale *Collegial nurse–physician relationship* (mean 3.24 [SD = 0.41]). The lowest mean score was observed for the subscale *Nurse participation in hospital affairs* (mean 2.53 [SD = 0.46]). All results are presented in Table 4.

**TABLE 4:** Experiences of ICU nurses towards a collaboration, staffing and the nursing work environment.

Practice environment scale of nursing work index (PES‐NWI)^∗^					
PES‐NWI total, mean (SD)	2.89 (0.31)	—			
Staffing and resource adequacy	2.95 (0.41)				
Collegial nurse–physician relationships	3.24 (0.41)				
Nurse manager ability, leadership and support of nurses	2.85 (0.53)				
Nursing foundation of quality of care	2.84 (0.33)				
Nurse participation in hospital affairs	2.53 (0.46)				

**PES-NWI Statements**	**Strongly disagree (%)**	**Disagree (%)**	**Agree (%)**	**Strongly agree (%)**	**Not applicable (%)**

Support services allow me to spend adequate time with my patients.	6 (10.3)	21 (36.2)	25 (43.1)	3 (5.2)	3 (5.2)
Physicians and nurses have good working relationships.	—	2 (3.4)	32 (55.2)	24 (41.4)	—
A supervisory staff that is supportive of the nurses.	2 (3.4)	10 (17.2)	36 (62.1)	9 (15.5)	1 (1.7)
Active staff development or continuing education programs for nurses.	2 (3.4)	7 (12.1)	36 (62.1)	12 (20.7)	1 (1.7)
Career development/clinical ladder opportunity.	1 (1.7)	8 (13.8)	34 (58.6)	15 (25.9)	—
Opportunity for staff nurses to participate in policy decisions.	14 (24.1)	29 (50.0)	12 (20.7)	3 (5.2)	—
Doctors value nurses’ observations and judgements.	—	1 (1.7)	39 (67.2)	17 (29.3)	1 (1.7)
Enough time and opportunity to discuss patient care problems with other nurses.	1 (1.7)	2 (3.4)	32 (55.2)	22 (37.9)	1 (1.7)
Enough registered nurses to provide quality patient care.	—	10 (17.2)	37 (63.8)	10 (17.2)	1 (1.7)
A nurse manager who is a good manager and leader.	2 (3.4)	4 (6.9)	40 (69.0)	10 (17.2)	2 (3.4)
Much teamwork between nurses and doctors.	—	4 (6.9)	19 (32.8)	35 (60.3)	—
Opportunities for career development/opportunities for advancement.	1 (1.7)	9 (15.5)	27 (46.6)	14 (24.1)	7 (12.1)
Enough staff to get the work done.	—	10 (17.2)	37 (63.8)	11 (19.0)	—
Praise and recognition for a job well done.	4 (6.9)	22 (37.9)	28 (48.3)	4 (6.9)	—
Working with nurses who are clinically competent.	—	1 (1.7)	38 (65.5)	17 (29.3)	2 (3.4)
High standard of nursing care is expected by the administration.	4 (6.9)	20 (34.5)	20 (34.5)	12 (20.7)	2 (3.4)
Doctors recognise the contribution of nurses to patient care/the contributions that nurses make to patient care are publicly acknowledged.	—	—	36 (62.1)	21 (36.2)	1 (1.7)
A chief nursing executive is equal in power and authority to other top‐level hospital officials.	10 (17.2)	23 (39.7)	11 (19.0)	5 (8.6)	9 (15.5)
Doctors respect nurses as equal professionals.	2 (3.4)	8 (13.8)	35 (60.3)	13 (22.4)	—
The environment in which patient care is provided is characterised by a clear nursing vision/primary nursing as the nursing delivery system.	—	19 (32.8)	30 (51.7)	8 (13.8)	1 (1.7)
A chief nursing officer is highly visible and accessible to staff.	5 (8.6)	15 (25.9)	24 (41.4)	9 (15.5)	5 (8.6)
An administration that listens and responds to employee concerns.	14 (24.1)	29 (50.0)	14 (24.1)	1 (1.7)	—
A nurse manager backs up the nursing staff in decision‐making, even if the conflict is with a physician.	2 (3.4)	11 (19.0)	30 (51.7)	10 (17.2)	5 (8.6)
(Staff) nurses are involved in the internal governance of the hospital (e.g. practice and policy committees).	15 (25.9)	25 (43.1)	15 (25.9)	1 (1.7)	2 (3.4)
Collaboration (joint practice) between nurses and physicians.	—	4 (6.9)	34 (58.6)	20 (34.5)	—
Nursing care is based on a nursing rather than a medical model.	1 (1.7)	24 (41.4)	29 (50.0)	2 (3.4)	2 (3.4)
Written, up‐to‐date nursing care plans for all patients.	4 (6.9)	25 (43.1)	23 (39.7)	2 (3.4)	4 (6.9)
A good orientation program for newly employed nurses.	1 (1.7)	13 (22.4)	40 (69.0)	3 (5.2)	1 (1.7)
(Staff) nurses have the opportunity to be part of hospital and nursing committees.	1 (1.7)	2 (3.4)	44 (75.86)	9 (15.5)	2 (3.4)
Patient assignments foster continuity of care (i.e. the same nurse cares for the patient from one day to the next).	—	6 (10.3)	41 (70.7)	11 (19.0)	—
An active quality‐assurance program.	—	14 (24.1)	37 (63.8)	5 (8.6)	2 (3.4)
Doctors have a high regard for nurses.	—	18 (31.0)	33 (56.9)	7 (12.1)	—

*Note:* Continuous data are given as mean (SD), unless reported as *n* (%).

^∗^The scale anchors ranged from 1 (strongly agree) to 4 (strongly disagree).

#### 3.3.2. Perceived Quality of Care and Collaboration

Three months after the integration, 58.5% (*n* = 34) of ICU nurses continued to express concerns about the quality of care (Figure 3), although the perceived quality of nursing care continued being assessed as an 8 [IQR 7 – 8]. Collaboration with care assistants was perceived positively by 82.8% (*n* = 48), and 51.7% (*n* = 30) indicated that care assistants were aware of their skills and permitted tasks related to ICU care (Figure 3). Among ICU nurses, a majority (*n* = 32, 55.2%) developed collaborative relationships with colleagues, including care assistants, and ensured that their work usually focused on achieving meaningful outcomes for patients (*n* = 32, 55.2%). Over half (*n* = 34, 58.6%) usually/almost always complimented and recognised the contributions of colleagues (Table 5). Care assistants rated their collaboration with ICU nurses as positive (*n* = 10, 90.9%), though five care assistants (45.5%) felt somewhat insecure in this relationship (Table 3). In addition, care assistants rated the perceived quality of care with an 8 out of 10 [IQR 8–9].

**TABLE 5:** Assessment of the nursing clinical leadership.

Clinical leadership scale(CLS)^∗^					
CLS total, sum score mean (SD)	61.33 (6.09)	—			
CLS total, composite mean score (SD)	4.09 (0.41)				
Challenging the process	4.19 (0.52)				
Inspiring a shared vision	4.07 (0.53)				
Enabling others to act	4.16 (0.52)				
Modelling the way	4.33 (0.44)				
Encouraging the heart	3.70 (0.74)				

**Self-perceived clinical leadership,** *n* (%)	**Disagree (%)**	**Agree (%)**	—		

I consider myself a clinical leader in my work.	31 (53.4)	27 (46.6)	—		
I exhibit leadership behaviour in my daily work.	13 (22.4)	45 (77.6)			

**CLS-statements**	**Almost never (%)**	**Sometimes (%)**	**Occasionally (%)**	**Usually (%)**	**Almost always (%)**

When I have concerns about my patient’s well‐being, I ask critical questions about assignments and/or treatments.	1 (1.7)	—	2 (3.4)	24 (41.4)	31 (53.4)
I support my clinical decisions with clinical reasoning and scientific insights.	—	5 (8.6)	6 (10.3)	33 (56.9)	14 (24.1)
I reflect on my own and others’ actions and strive to understand what went well and what did not.	—	2 (3.4)	3 (5.2)	37 (63.8)	16 (27.6)
I negotiate with and support direct colleagues and colleagues from other disciplines to help patients achieve their personal goals.	—	1 (1.7)	3 (5.2)	39 (67.2)	15 (25.9)
I feel engaged with patients when communicating with them about achieving personal goals.	1 (1.7)	—	7 (12.1)	34 (58.6)	16 (27.6)
I conduct meaningful conversations with direct colleagues and colleagues from other disciplines to ensure we provide better, safer and more person‐centred care.	—	—	17 (29.3)	28 (48.3)	13 (22.4)
I actively listen to the different opinions and perspectives of direct colleagues and colleagues from other disciplines.	—	—	7 (12.1)	36 (62.1)	15 (25.9)
I build a therapeutic relationship with patients and their family members based on mutual trust and respect.	—	—	7 (12.1)	31 (53.4)	20 (34.5)
I develop collaborative relationships with my direct colleagues and colleagues from other disciplines, such as care assistants.	—	4 (6.9)	4 (6.9)	32 (55.2)	18 (31.0)
I fulfil my promises and obligations to patients.	—	—	—	26 (44.8)	32 (55.2)
I ensure that we work towards achievable goals with measurable objectives to achieve clinical outcomes for patients.	1 (1.7)	2 (3.4)	7 (12.1)	32 (55.2)	16 (27.6)
I provide person‐centred care.	—	—	1 (1.7)	32 (55.2)	25 (43.1)
I publicly compliment direct colleagues and colleagues from other disciplines who are role models for professionalism.	1 (1.7)	2 (3.4)	24 (41.4)	21 (36.2)	10 (17.2)
I give positive feedback to direct colleagues and colleagues from other disciplines who contribute to the well‐being of patients and their families through their actions.	1 (1.7)	—	23 (39.7)	26 (44.8)	8 (13.8)
I always find ways to show appreciation for the achievements of direct colleagues and colleagues from other disciplines.	—	2 (3.4)	18 (31.0)	30 (51.7)	8 (13.8)

*Note:* Continuous data are given as mean (SD), unless reported as *n* (%).

^∗^The scale anchors ranged from 1 (almost never) to 5 (almost always).

#### 3.3.3. Clinical Leadership

The total leadership scores ranged from 50 to 75, with a mean of 61.33 (SD = 6.09), reflecting moderate to high levels of leadership behaviour. The overall CLS composite mean score was 4.09 (SD = 0.41), indicating a high level of perceived clinical leadership among participants. Among the five subscales, the highest mean score was observed for *Modelling the way* (4.33 [SD = 0.44]). The lowest mean score was found for *Encouraging the heart* (3.70 [SD = 0.74]). In addition, ICU nurses reported leadership behaviour in their daily work (*n* = 45, 77.6%), yet they did not see themselves as a clinical leader (*n* = 31, 53.4%). ICU nurses indicated strong engagement with both colleagues and patients and predominantly responded ‘Usually’ and ‘Almost always’ across the different aspects of clinical leadership. All results are presented in Table 5.

#### 3.3.4. Work Satisfaction and Intention to Leave

Nearly all ICU nurses reported high job satisfaction at both time points: before the introduction of care assistants (*n* = 91, 95.8%) and after (*n* = 56, 96.6%). Most ICU nurses would recommend the hospital as a workplace to colleagues (before: *n* = 83, 87.4%; and after: *n* = 46, 79.3%. In addition, 89.7% (*n* = 52) of ICU nurses were not considering leaving their job in the next year, although most acknowledged that it would be easy (*n* = 22, 37.9%) to very easy (*n* = 33, 56.9%) to find an acceptable alternative job. Care assistants were satisfied with their work in the ICU, and none expressed an intention to leave the ICU as a workplace.

#### 3.3.5. ICU Nurses’ Narratives of Their Experiences With Integrated Teams

Several ICU nurses described mixed experiences regarding leadership and managerial support during the integration of care assistants. While management was perceived as engaged, participants emphasised the need for clearer guidance and role clarification. As one nurse noted, ‘*In terms of leadership, I think they could do more to clarify what is expected from the care assistants and how they can best integrate’.* Others highlighted that responsibility for successful integration also lay with ICU nurses themselves: ‘*It’s not so much about the manager; the ICU nurses themselves need to put in time and energy to integrate the care assistants properly’.* ICU nurses further emphasised the need for ongoing supervision and training of care assistants. One respondent remarked that ‘*Even after 3 months, care assistants still don’t fully understand their tasks, and it’s unclear what their responsibilities are’.*(See Supporting File 2 for additional comments)

#### 3.3.6. Care Assistants’ Narratives of Their Experiences With Integrated Teams

Care assistants (*n* = 5, 45.5%) expressed confidence in their ability to handle daily tasks, such as assisting with fundamental care. One care assistant highlighted: ‘*The tasks I perform go well, and I am allowed to voice discomfort if needed’.* However, another care assistant indicated varying levels of comfort, noting that she was still adjusting to the ICU environment: ‘*It varies per day, especially as I am still getting used to the ICU’.* Several care assistants expressed a desire to re‐evaluate the scope of their role, indicating that their current responsibilities did not fully align with their qualifications. One respondent stated, ‘*According to our qualifications, we should be allowed to do much more than what we are currently permitted to do on this ICU; the scope of permitted tasks should be reviewed’.* The majority found collaboration with ICU nurses positive, emphasising the support they received from the nurses, for example, ‘*The more we work together, the more my help is requested’* and ‘*Collaboration generally goes smoothly’*. However, some noted a lack of involvement in direct care tasks, for example, ‘*Sometimes the ICU nurses prefer to do tasks themselves and don’t call me to assist, which might be a trust issue’* and ‘*One nurse communicates with you and makes you feel valued, while another almost ignores you. This makes you feel lost and lonely’.* Others indicated that improved communication and clearer task division could enhance team dynamics: ‘*More attention to communication during handovers could make teamwork smoother.’*

## 4. Discussion

Undertaking a model of care with an integrated team of ICU nurses and care assistants represents a potential strategy to address the pressing issues of nursing shortages and high workloads within the ICU. This study shows that, although both ICU nurses and care assistants initially expected such models to be useful, persistent challenges related to collaboration and clinical leadership hindered improvements in perceived quality of patient care and positive professional experiences.

These results indicate that integration of care assistants into ICU nursing teams requires a fundamental shift in the way ICU professionals collaborate, rather than merely adding staff to the existing care models. Successful implementation depends on clear expectations and coordinated adjustments at multiple levels, including individual professionals’ team leadership and hospital management. A skill mix in the ICU is therefore not a one‐size‐fits‐all solution; it requires structural, cultural and educational adaptations to ensure effective collaboration while maintaining high standards of patient care.

ICU nurses expected the introduction of care assistants to provide a valuable expansion of the nursing team, recognising the potential benefits of incorporating professionals with different educational backgrounds. Care assistants reported being positive about their integration and felt welcome in the team. Notably, the majority of ICU nurses hesitated to endorse the initiative and indicated that it should not be continued. The consistently high job satisfaction among ICU nurses, along with the positively perceived nursing work environment, suggests that the rejection of a skill mix within the team was not driven by dissatisfaction with the work environment or their own job satisfaction.

### 4.1. Challenging Role Clarities, Touching on Collaboration

Both ICU nurses and care assistants experienced their collaboration as positive and cohesive, although care assistants expressed uncertainty about their role. This insecurity seemed to continue through a lack of role clarity; care assistants understood their responsibilities and tasks—despite having a more restricted scope of practice in the ICU compared to other healthcare settings—while ICU nurses continued to wonder about the permitted tasks of care assistants. Previous studies showed that the integration of care assistants into ICU teams and the creation of new roles within critical care settings were intended to manage patient care and support ICU nurses, rather than replace them [17]. While the shortage of qualified ICU nurses and the aims of cost containment are major drivers for employing care assistants, it remained essential to view care assistants as complementary to ICU nurses rather than as substitutes [16, 17]. Concurrently, employing unregistered staff or care assistants could have a positive impact on patient care, leading to more timely nursing interventions, improved patient rehabilitation and enhanced communication among all stakeholders [11]. To make a skill mix work effectively in the ICU setting, it is crucial to invest in clearly defined roles, ongoing interdisciplinary communication and shared understanding of responsibilities.

### 4.2. Quality of Care as a Core Aspect of Essential Nursing Care

Both ICU nurses and care assistants perceived the quality of care as high at both measurements. However, post‐integration, ICU nurses expressed ongoing concerns regarding patient safety and overall care quality. While these concerns coexisted with consistently high quality ratings, ICU nurses reported that staffing levels remained sufficient to deliver high‐quality patient care. This may partly explain why perceived care quality did not decline, despite hesitation around delegation and concerns about patient safety. A recent systematic review on care assistants, although not specific to the ICU setting, reports similarly mixed findings: some studies suggest that their involvement can enhance care quality by enabling nurses to focus on high‐acuity and critical care activities, whereas others identify potential risks to patient safety in cases of unclear role definitions, inadequate training, or lack of supervision [53]. In practice, care assistants often perform a broad range of fundamental nursing tasks that—when supported by competency‐based training and appropriate supervision—can meaningfully contribute to patient care [53].

One of the anticipated benefits of integrating care assistants was an increase in perceived staffing and resource adequacy. The results indicated that ICU nurses did not experience a differed workload, nor did they get additional time to deliver complex ICU care—a contrast to what is commonly reported in the literature [11, 54, 55]. Several factors may explain this discrepancy. First, care assistants may be limited in taking over tasks sufficiently to reduce ICU nurses′ workload. Although care assistants were assigned to a broad set of responsibilities and knew which tasks they were permitted to carry out, they were still becoming accustomed to the ICU environment. Second, ICU nurses may have felt a strong sense of responsibility over patient care, leading them to retain control over certain tasks rather than delegate them to care assistants [16, 55]. While care assistants were expected to perform both routine and fundamental nursing tasks [55], the ICU nurses may have hesitated to relinquish control over patient care due to concerns about losing direct oversight. This reluctance to delegate is consistent with findings by Crevacore et al., who reported that ICU nurses prefer to maintain first‐hand knowledge of their patients′ conditions, fearing that delegation may result in a loss of control over clinical decision‐making [16]. This may impact the quality of fundamental nursing care.

### 4.3. Recognising Clinical Leadership Among ICU Nurses, Including the Entanglement of Holistic Nursing Tasks

While differences in educational backgrounds between ICU nurses and care assistants present challenges, they also provide opportunities for ICU nurses to demonstrate and develop their clinical leadership skills. By taking on a coordinating role, ICU nurses could enhance their professional autonomy and clinical leadership skills [7]. With high‐tech care delivery and advanced medical treatments in life‐threatening health situations in the ICU, clinical nursing leadership needs critical capacity and autonomy to provide effective interventions in situations involving acutely deteriorating patients [56]. The findings of this study confirm that the majority of ICU nurses self‐reported exhibiting leadership behaviour in their daily practice, as reflected in their high overall scores on the CLS, with all subscales exceeding the threshold for positive clinical leadership. The highest‐scoring subscale, modelling the way, indicates a strong sense of professional responsibility, which may also explain nurses’ reluctance to delegate patient‐related tasks they perceive as central to their role. Notably, they did not perceive themselves as clinical leaders. Sharing responsibilities, particularly in a high‐acuity setting such as the ICU, requires a high level of clinical leadership [7, 29]. However, the findings suggest that ICU nurses may struggle with delegating critical aspects of patient care to newly integrated care assistants who are not yet fully trained in the ICU context [16].

This discrepancy between demonstrated leadership behaviours and the absence of self‐identification as a clinical leader illustrates how leadership can be practiced without being explicitly recognised as part of one’s professional role. This tension is closely related to the professional identity as ICU nurses often associate their role with maintaining control over core aspects of patient care, reinforcing responsibility and autonomy. While such control is understandable in high‐risk settings, it may generate resistance when established workflows are disrupted or when collaboration with less experienced or differently trained colleagues is required. In this context, delegating tasks to newly integrated care assistants can evoke uncertainty and challenge existing routines. Although ICU nurses currently perceive themselves as capable of managing all aspects of care independently, this approach may prove unsustainable given of projected nursing workforce shortages. Future practice will therefore require greater trust in the competencies of care assistants and a willingness to adopt new collaborative models, alongside increased flexibility in how ICU nurses define and enact their professional identity. Another key factor influencing the introduction of skill mix and role differentiation is the holistic and inherently entangled nature of ICU nursing care [22, 57]. ICU nurses do not perform isolated tasks; rather, nursing activities are interwoven, meaning that even seemingly routine actions, such as hygiene care, are closely connected to physiological monitoring, decision‐making, and relational aspects of care. This entanglement makes delegation particularly challenging, as separating tasks may alter their clinical meaning and impact. Routine care activities provide ICU nurses with critical opportunities for ongoing patient assessment, early detection of deterioration, and therapeutic interaction. The findings of this study support the theoretical perspective that nursing care in the ICU should not be fragmented into discrete tasks but instead organised to preserve continuity and integration across different provider roles [22].

### 4.4. Strengths and Limitations

A key strength of this study is its multimethod approach, which combines both quantitative and qualitative data to provide a comprehensive understanding of ICU nurses′ and care assistants′ expectations and experiences within an integrated team. This design offers rich contextual insights into the implementation process. Moreover, a large amount of data have been gathered in a relatively short study period to gain valuable understanding of the complex process of forming integrated teams with different roles and skills. Despite these strengths, several limitations should be acknowledged. First, variables such as clinical leadership and the nursing work environment were not assessed at baseline, making it impossible to measure changes over time. Although these variables scored highly, it remains unclear whether they were already high before the intervention, making it difficult to evaluate the true impact of care assistant integration. Second, potential nonresponse bias must be considered, as a notable difference in response rates was observed between both time points. This was likely influenced by the length of the survey, which may have contributed to survey fatigue. In addition, it is possible that respondents with stronger opinions—either positive or negative—were more likely to complete the follow‐up questionnaire, which may have affected the representativeness of the results. As such, the findings from the second measurement point should be interpreted with appropriate caution. Third, this study was conducted in two ICU departments within a tertiary academic centre, which may limit the generalisability of the findings to other hospital settings or nonacademic ICUs. Caution is required when applying these results to different healthcare environments. Fourth, data on care assistants’ experiences were only collected after three months of employment, without a baseline measurement. Further research is needed to better understand their expectations, attitudes, integration, and professional experiences over time. Fifth, the relatively short study period may have limited our ability to fully assess whether the integration of care assistants was successful. Although a slight majority of ICU nurses indicated that the initiative should be discontinued, this finding should be interpreted with caution. Team integration is a complex and dynamic process that typically requires more time for proper evaluation and adaptation. Therefore, it is possible that some of the longer‐term effects were not yet visible within the timeframe of this study.

### 4.5. Future Lines of Research

Future studies should focus on the long‐term effects of integrating care assistants in the ICU environment, particularly regarding overall quality of care, patient safety, and cost‐effectiveness [58, 59]. While previous research has explored the role of care assistants, more empirical studies—especially longitudinal research—are needed to assess whether a skill mix of various health professionals other than registered nurses enhances or compromises clinical outcomes [60]. To better understand the implications of skill mixing in ICU teams, future research should also focus on its long‐term effects on ICU nursing professionals, including workload, job satisfaction, and intention to leave the profession [61]. These factors are crucial for ensuring workforce sustainability and resilient ICU teams, as excessive workloads and dissatisfaction contribute to burnout, turnover, and staffing shortages, which in turn can affect the quality and continuity of patient care. Another key area for future research is clinical leadership, autonomy, and delegation of roles among ICU nurses. Developing targeted interventions to support ICU nurses in strengthening their leadership and delegation abilities may facilitate more effective collaboration with care assistants while maintaining high standards of patient care.

### 4.6. Practical Implications

Based on the findings of this study, several recommendations can be formulated to support the effective and sustainable integration of care assistants into ICU nursing teams.

#### 4.6.1. Clarify Roles and Tasks

Clear delineation of tasks and responsibilities is essential to align the care assistant’s role with their competencies and to reduce uncertainty among ICU nurses. Shared competency frameworks can support transparency and mutual understanding within the team [62].

#### 4.6.2. Invest in Targeted Training and Education

Care assistants working in the ICU require specialised training tailored to the critical care context, including emergency procedures, resuscitation protocols and fundamental care for complex ICU patients, to ensure safe task execution [12].

#### 4.6.3. Strengthen Collaboration and Communication

Involving care assistants in team‐based processes such as patient handovers, interprofessional training and simulation exercises can enhance communication, trust and team integration [63].

#### 4.6.4. Support Safe Delegation and Clinical Leadership

Effective delegation requires that ICU nurses are supported with clear guidelines, leadership encouragement and confidence in the competencies of care assistants, ensuring that delegated tasks remain within regulatory and professional boundaries.

#### 4.6.5. Implement Structured Onboarding and Evaluation

A comprehensive onboarding program, combined with ongoing feedback and reflection, is crucial to establish role clarity, support collaboration and maintain a sustainable skill mix over time [64].

## 5. Conclusions

This study provides insights into the reorganisation of nursing care through the integration of a skill mix in ICU teams. A care model integrating ICU nurses and care assistants initially received positive engagement from both groups. ICU nurses viewed the introduction of care assistants as a potential expansion of workforce capacity, with anticipated benefits for workload distribution and patient care, while care assistants felt welcomed within the ICU environment. Over time, however, this initial enthusiasm diminished as practical challenges emerged. Persistent concerns regarding care quality and patient safety remained, alongside mismatches between care assistants’ competencies and the demands of the ICU environment.

The introduction of an integrated ICU nursing team is insufficient as a standalone response to nursing shortages. Effective role delineation and collaboration are fundamental to optimising care delivery within skill mixed teams. This study highlights the complexity of skill mix implementation in the ICU and emphasises the need to balance task delegation with professional development to support safe and sustainable care models.

## Author Contributions

Atreyu van Esch: conceptualization, investigation, data curation, formal analysis, validation, writing–original draft and project administration. Dewi Stalpers: validation and writing–review and editing. Margo M. C. van Mol: conceptualization, investigation, validation, writing original draft–review and editing and supervision.

## Funding

This research did not receive any specific grants from funding agencies.

## Ethics Statement

The Medical Ethics Committee of the Erasmus University Medical Center Rotterdam granted approval for the study (MEC‐2023‐0551). The committee evaluated the research proposal and determined that the requirements outlined in the Dutch medical research involving human subjects Act were not applicable to the current study. It was conducted in adherence to the ethical principles delineated in the Declaration of Helsinki (75th WMA General Assembly, Fortaleza, Helsinki, October 2024) and according to good clinical practice guidelines. Both oral and written informed consent were obtained from all participants. They were informed of their right to withdraw from the study at any time. The collected data were processed in a pseudo anonymous manner to ensure the participants’ privacy.

## Conflicts of Interest

The authors declare no conflicts of interest.

## Supporting Information

Additional supporting information can be found online in the Supporting Information section.

## Supporting information


**Supporting Information 1** Supporting File 1. Complete questionnaires 1, 2 and 3.

**Supporting Information 2** Supporting File 2. Narratives and expectations of ICU nurses.

## Data Availability

The data that support the findings of this study are available on request from the corresponding author. The data are not publicly available due to privacy or ethical restrictions.

## References

[1] Tamata A. T. and Mohammadnezhad M. , A Systematic Review Study on the Factors Affecting Shortage of Nursing Workforce in the Hospitals, Nursing Open. (2023) 10, no. 3, 1247–1257, 10.1002/nop2.1434. 36303066 PMC9912424

[2] Bae S. H. , Noneconomic and Economic Impacts of Nurse Turnover in Hospitals: A Systematic Review, International Nursing Review. (2022) 69, no. 3, 392–404, 10.1111/inr.12769. 35654041 PMC9545246

[3] Stechmiller J. K. , The Nursing Shortage in Acute and Critical Care Settings, AACN Advanced Critical Care. (2002) 13, no. 4, 577–584, 10.1097/00044067-200211000-00011, 2-s2.0-0036832197. 12473920

[4] International Council of Nurses , ICN Policy Brief: The Global Nursing Shortage and Nurse Retention, 2021, Geneva, Switzerland.

[5] Dean W. et al., Moral Injury and the Global Health Workforce Crisis—Insights from an International Partnership, New England Journal of Medicine. (2024) . 10.1056/NEJMp2402833 39216090

[6] Vincent J.-L. , Boulanger C. , van Mol M. M. C. , Hawryluck L. , and Azoulay E. , Ten Areas for ICU Clinicians to be Aware of to Help Retain Nurses in the ICU, Critical Care. (2022) 26, no. 1, 10.1186/s13054-022-04182-y. PMC9559151 36229859

[7] Stalpers D. , Tilburgs B. , and van Mol M. , Take Control by Letting Go? Sustainable Employability of Nurses in Intensive Care Units, Nursing in Critical Care. (2021) . 10.1111/nicc.12725 34647395

[8] de Vries N. , Boone A. , Godderis L. et al., The Race to Retain Healthcare Workers: A Systematic Review on Factors that Impact Retention of Nurses and Physicians in Hospitals, Inquiry: The Journal of Health Care Organization, Provision, and Financing. (2023) 60, 10.1177/00469580231159318. PMC10014988 36912131

[9] Geltmeyer K. , Eeckloo K. , Dehennin L. et al., How Much do We Know About Nursing Care Delivery Models in a Hospital Setting? A Mapping Review, Nursing Inquiry. (2024) 31, no. 3, 10.1111/nin.12636. 38536152

[10] Buchan J. and Dal Poz M. R. , Skill Mix in the Health Care Workforce: Reviewing the Evidence, Bulletin of the World Health Organization. (2002) 80, no. 7, 575–580. 12163922 PMC2567564

[11] McGuire A. , Richardson A. , Coghill E. , Platt A. , Wimpenny S. , and Eglon P. , Implementation and Evaluation of the Critical Care Assistant Role, Nursing in Critical Care. (2007) 12, no. 5, 242–249, 10.1111/j.1478-5153.2007.00230.x, 2-s2.0-38449111464. 17883617

[12] Sutton J. , Valentine J. , and Rayment K. , Staff Views on the Extended Role of Health Care Assistants in the Critical Care Unit, Intensive and Critical Care Nursing. (2004) 20, no. 5, 249–256, 10.1016/j.iccn.2004.06.006, 2-s2.0-4644300777. 15450613

[13] Bruyneel L. , Li B. , Aiken L. , Lesaffre E. , Van den Heede K. , and Sermeus W. , A multi-country Perspective on Nurses’ Tasks Below Their Skill Level: Reports from Domestically Trained Nurses and Foreign Trained Nurses from Developing Countries, International Journal of Nursing Studies. (2013) 50, no. 2, 202–209, 10.1016/j.ijnurstu.2012.06.013, 2-s2.0-84872374443. 22819343

[14] Bohlinger S. , Ten Years After: The ‘Success Story’ of the European Qualifications Framework, Journal of Education and Work. (2019) 32, no. 4, 393–406, 10.1080/13639080.2019.1646413, 2-s2.0-85070290746.

[15] Feo R. and Kitson A. , Promoting patient-centred Fundamental Care in Acute Healthcare Systems, International Journal of Nursing Studies. (2016) 57, 1–11, 10.1016/j.ijnurstu.2016.01.006, 2-s2.0-84960943715. 27045560

[16] Crevacore C. , Jacob E. , Coventry L. L. , and Duffield C. , Integrative Review: Factors Impacting Effective Delegation Practices by Registered Nurses to Assistants in Nursing, Journal of Advanced Nursing. (2023) 79, no. 3, 885–895, 10.1111/jan.15430. 36062891

[17] Twigg D. E. , Myers H. , Duffield C. , Pugh J. D. , Gelder L. , and Roche M. , The Impact of Adding Assistants in Nursing to Acute Care Hospital Ward Nurse Staffing on Adverse Patient Outcomes: An Analysis of Administrative Health Data, International Journal of Nursing Studies. (2016) 63, 189–200, 10.1016/j.ijnurstu.2016.09.008, 2-s2.0-84988322481. 27653280

[18] Wainwright T. A. , The Perceived Function of Health Care Assistants in Intensive Care: Nurses Views, Intensive and Critical Care Nursing. (2002) 18, no. 3, 171–180, 10.1016/s0964-3397(02)00027-7, 2-s2.0-0036592982. 12405272

[19] Carroll R. E. , De Vries K. , Goodman C. , and Brown J. , Health Care Assistant and Registered Nurse Dyads, Working Together and Apart–A Qualitative Study, BMC Nursing. (2024) 23, no. 1, 10.1186/s12912-024-02619-z. PMC11687144 39736672

[20] Pattison N. , An ever-thorny Issue: Defining Key Elements of Critical Care Nursing and its Relation to Staffing, Nursing in Critical Care. (2021) . 10.1111/nicc.12726 34783138

[21] San Juan N. V. , Clark S. E. , Camilleri M. et al., Training and Redeployment of Healthcare Workers to Intensive Care Units (ICUs) During the COVID-19 Pandemic: A Systematic Review, BMJ Open. (2022) 12, no. 1, 10.1136/bmjopen-2021-050038. PMC8753114 34996785

[22] Stalpers D. , Schoonhoven L. , Dall’Ora C. , Ball J. , and Griffiths P. , Entanglement of Nursing Care’: A Theoretical Proposition to Understand the Complexity of Nursing Work and Division of Labour, International Journal of Nursing Studies. (2025) 163, 10.1016/j.ijnurstu.2025.104995. 39818169

[23] Ross P. , Jaspers R. , Watterson J. et al., The Impact of Nursing Workforce skill-mix on Patient Outcomes in Intensive Care Units in Victoria, Australia, Critical Care and Resuscitation. (2024) 26, no. 2, 135–152, 10.1016/j.ccrj.2024.03.002. 39072235 PMC11282374

[24] Twigg D. E. , Kutzer Y. , Jacob E. , and Seaman K. , A Quantitative Systematic Review Of The Association Between Nurse Skill Mix And Nursing-Sensitive Patient Outcomes In The Acute Care Setting, Journal of Advanced Nursing. (2019) 75, no. 12, 3404–3423, 10.1111/jan.14194, 2-s2.0-85073975241. 31483509 PMC6899638

[25] Griffiths P. , Maruotti A. , Recio Saucedo A. et al., Nurse Staffing, Nursing Assistants and Hospital Mortality: Retrospective Longitudinal Cohort Study, BMJ Quality and Safety. (2019) 28, no. 8, 609–617, 10.1136/bmjqs-2018-008043, 2-s2.0-85058098590. PMC6716358 30514780

[26] Iraizoz-Iraizoz A. , García-García R. , Navarrete-Muro A. , Blasco-Zafra A. , Rodríguez-Beperet A. , and Vázquez-Calatayud M. , Nurses’ Clinical Leadership in the Intensive Care Unit: A Scoping Review, Intensive and Critical Care Nursing. (2023) 75, 10.1016/j.iccn.2022.103368. 36528457

[27] James A. H. , Bennett C. L. , Blanchard D. , and Stanley D. , Nursing and Values‐Based Leadership: A Literature Review, Journal of Nursing Management. (2021) 29, no. 5, 916–930, 10.1111/jonm.13273. 33484188

[28] Huang H. , Zhang X. , Tu L. et al., Inclusive Leadership, Self-Efficacy, Organization-Based Self-Esteem, And Intensive Care Nurses’ Job Performance: A Cross-Sectional Study Using Structural Equation Modeling, Intensive and Critical Care Nursing. (2024) 87, 10.1016/j.iccn.2024.103880. 39500700

[29] Cummings G. G. , Lee S. , Tate K. et al., The Essentials of Nursing Leadership: A Systematic Review of Factors and Educational Interventions Influencing Nursing Leadership, International Journal of Nursing Studies. (2021) 115, 10.1016/j.ijnurstu.2020.103842. 33383271

[30] Van Kraaij J. , Lalleman P. , Walravens A. , and Van Oostveen C. , Differentiated Nursing Practice as a Catalyst for Transformations in Nursing: A Multiphase Qualitative Interview Study, Journal of Advanced Nursing. (2022) 78, no. 1, 165–175, 10.1111/jan.15001. 34375011 PMC9292649

[31] Li T. M. , Pien L. , Kao C. , Kubo T. , and Cheng W. , Effects of Work Conditions and Organisational Strategies on Nurses’ Mental Health During the COVID‐19 Pandemic, Journal of Nursing Management. (2022) 30, no. 1, 71–78, 10.1111/jonm.13485. 34590379 PMC8646663

[32] Martini D. , Schalkwijk H. , Schoonhoven L. , Noordegraaf M. , and Lalleman P. , Working on Differentiated Nursing Practices in Hospitals: A Learning History on Enacting New Nursing Roles, Journal of Advanced Nursing. (2025) 81, no. 1, 439–449, 10.1111/jan.16240. 38808499 PMC11638512

[33] Bruyneel A. , Bouckaert N. , Pirson M. , Sermeus W. , and Van den Heede K. , Unfinished Nursing Care in Intensive Care Units and the Mediating Role of the Association Between Nurse Working Environment, and Quality of Care and Nurses’ Wellbeing, Intensive and Critical Care Nursing. (2024) 81, 10.1016/j.iccn.2023.103596. 38043435

[34] Dilig-Ruiz A. , MacDonald I. , Demery Varin M. , Vandyk A. , Graham I. D. , and Squires J. E. , Job Satisfaction Among Critical Care Nurses: A Systematic Review, International Journal of Nursing Studies. (2018) 88, 123–134, 10.1016/j.ijnurstu.2018.08.014, 2-s2.0-85054320850. 30292878

[35] Vleminckx S. , Van Bogaert P. , De Meulenaere K. , Willem L. , and Haegdorens F. , Factors Influencing the Formation of Balanced Care Teams: The Organisation, Performance, and Perception of Nursing Care Teams and the Link with Patient Outcomes: A Systematic Scoping Review, BMC Health Services Research. (2024) 24, no. 1, 10.1186/s12913-024-11625-5. PMC11429156 39334182

[36] Anguera M. T. , Blanco-Villaseñor A. , Losada J. L. , Sánchez-Algarra P. , and Onwuegbuzie A. J. , Revisiting the Difference Between Mixed Methods and Multimethods: Is it all in the Name?, Quality and Quantity. (2018) 52, no. 6, 2757–2770, 10.1007/s11135-018-0700-2, 2-s2.0-85041836501.

[37] Schoonenboom J. and Johnson R. B. , How to Construct a Mixed Methods Research Design, Kolner Zeitschrift fur Soziologie und Sozialpsychologie. (2017) 69, no. Suppl 2, 107–131, 10.1007/s11577-017-0454-1, 2-s2.0-85029628651. 28989188 PMC5602001

[38] Salimi-Bani M. , Pandian V. , Vahedian-Azimi A. , Moradian S. T. , and Bahramifar A. , A Respiratory Critical Care Nurse Training Program for Settings Without a Registered Respiratory Therapists: A Protocol for a Multimethod Study, Intensive and Critical Care Nursing. (2024) 82, 10.1016/j.iccn.2024.103662. 38382240

[39] Acevedo-Nuevo M. , González-Gil M. T. , and Martin-Arribas M. C. , Physical Restraint Use in Intensive Care Units: Exploring the decision-making Process and New Proposals. A Multimethod Study, International Journal of Environmental Research and Public Health. (2021) 18, no. 22, 10.3390/ijerph182211826. PMC8623552 34831583

[40] Von Elm E. , Altman D. G. , Egger M. , Pocock S. J. , Gøtzsche P. C. , and Vandenbroucke J. P. , The Strengthening the Reporting of Observational Studies in Epidemiology (STROBE) Statement: Guidelines for Reporting Observational Studies, International Journal of Surgery. (2014) 12, no. 12, 1495–1499, 10.1016/j.ijsu.2014.07.013, 2-s2.0-84919384602. 25046131

[41] Tong A. , Sainsbury P. , and Craig J. , Consolidated Criteria for Reporting Qualitative Research (COREQ): A 32-item Checklist for Interviews and Focus Groups, International Journal for Quality in Health Care. (2007) 19, no. 6, 349–357, 10.1093/intqhc/mzm042, 2-s2.0-36549063576. 17872937

[42] Maassen S. M. , Weggelaar Jansen A. M. J. W. , Brekelmans G. , Vermeulen H. , and van Oostveen C. J. , Psychometric Evaluation of Instruments Measuring the Work Environment of Healthcare Professionals in Hospitals: A Systematic Literature Review, International Journal for Quality in Health Care. (2020) 32, no. 8, 545–557, 10.1093/intqhc/mzaa072. 32648902 PMC7654380

[43] Maassen S. M. , van Oostveen C. , Vermeulen H. , and Weggelaar A. M. , Defining a Positive Work Environment for Hospital Healthcare Professionals: A Delphi Study, PLoS One. (2021) 16, no. 2, 10.1371/journal.pone.0247530. PMC7906333 33630923

[44] Lake E. T. , Development of the Practice Environment Scale of the Nursing Work Index, Research in Nursing & Health. (2002) 25, no. 3, 176–188, 10.1002/nur.10032, 2-s2.0-0036615403. 12015780

[45] Sermeus W. , Aiken L. H. , Van den Heede K. et al., Nurse Forecasting in Europe (RN4CAST): Rationale, Design and Methodology, BMC Nursing. (2011) 10, no. 1, 10.1186/1472-6955-10-6, 2-s2.0-79955107761. PMC3108324 21501487

[46] Patrick A. , Laschinger H. K. S. , Wong C. , and Finegan J. , Developing and Testing a New Measure of Staff Nurse Clinical Leadership: The Clinical Leadership Survey, Journal of Nursing Management. (2011) 19, no. 4, 449–460, 10.1111/j.1365-2834.2011.01238.x, 2-s2.0-79955977124. 21569142

[47] Braam A. , van Wijngaarden J. D. H. , Vollmann M. , Hilders C. G. J. M. , and Buljac-Samardžić M. , Clinical Leaders Crossing Boundaries: A Study on the Role of Clinical Leadership in Crossing Boundaries Between Specialties, PLoS One. (2023) 18, no. 11, 10.1371/journal.pone.0294264. PMC10635562 37943885

[48] Karasek R. , Brisson C. , Kawakami N. , Houtman I. , Bongers P. , and Amick B. , The Job Content Questionnaire (JCQ): An Instrument for Internationally Comparative Assessments of Psychosocial Job Characteristics, Journal of Occupational Health Psychology. (1998) 3, no. 4, 322–355, 10.1037/1076-8998.3.4.322, 2-s2.0-0032177970. 9805280

[49] Mateen B. A. , Doogan C. , Hayward K. , Hourihan S. , Hurford J. , and Playford E. D. , Systematic Review of health-related Work Outcome Measures and Quality Criteria-based Evaluations of Their Psychometric Properties, Archives of Physical Medicine and Rehabilitation. (2017) 98, no. 3, 534–560, 10.1016/j.apmr.2016.06.013, 2-s2.0-85009205024. 27424293

[50] McNall M. and Foster-Fishman P. G. , Methods of Rapid Evaluation, Assessment, and Appraisal, American Journal of Evaluation. (2007) 28, no. 2, 151–168, 10.1177/1098214007300895, 2-s2.0-34249038901.

[51] McMullen C. K. et al., Rapid Assessment of Clinical Information Systems in the Healthcare Setting, Methods of Information in Medicine. (2011) 50, no. 04, 299–307. 21170469 10.3414/ME10-01-0042 PMC3746487

[52] Braun V. and Clarke V. , Using Thematic Analysis in Psychology, Qualitative Research in Psychology. (2006) 3, no. 2, 77–101, 10.1191/1478088706qp063oa, 2-s2.0-33750505977.

[53] Kagonya V. A. , Onyango O. O. , Maina M. , Gathara D. , English M. , and Imam A. , Characterising Support and Care Assistants in Formal Hospital Settings: A Scoping Review, Human Resources for Health. (2023) 21, no. 1, 10.1186/s12960-023-00877-7. PMC10680191 38012737

[54] Wilson N. J. , Pracilio A. , Morphet J. et al., A Scoping Review of Registered Nurses’ Delegating Care and Support to Unlicenced Care and Support Workers, Journal of Clinical Nursing. (2023) 32, no. 17-18, 6000–6011, 10.1111/jocn.16724. 37149737

[55] Wong K. L. , Chua W. L. , Griffiths P. et al., Teamwork Between Registered Nurses and Unlicensed Assistive Personnel in Acute Care Settings: A Scoping Review, International Journal of Nursing Studies Advances. (2025) 8, 10.1016/j.ijnsa.2025.100293. PMC11791319 39906753

[56] Brewster D. J. , Butt W. W. , Gordon L. J. , and Rees C. E. , Leadership in Intensive Care: A Review, Anaesthesia & Intensive Care. (2020) 48, no. 4, 266–276, 10.1177/0310057x20937319. 32741196

[57] Thornton L. , A Brief History and Overview of Holistic Nursing, Integrative Medicine: A Clinician’s Journal. (2019) 18, no. 4, 32–33. PMC7219452 32549829

[58] Griffiths P. , Recio‐Saucedo A. , Dall’Ora C. et al., The Association Between Nurse Staffing and Omissions in Nursing Care: A Systematic Review, Journal of Advanced Nursing. (2018) 74, no. 7, 1474–1487, 10.1111/jan.13564, 2-s2.0-85048874913. 29517813 PMC6033178

[59] Griffiths P. , Saville C. , Ball J. et al., Costs and cost-effectiveness of Improved Nurse Staffing Levels and Skill Mix in Acute Hospitals: A Systematic Review, International Journal of Nursing Studies. (2023) 147, 10.1016/j.ijnurstu.2023.104601. 37742413

[60] Dall’Ora C. , Saville C. , Rubbo B. , Turner L. , Jones J. , and Griffiths P. , Nurse Staffing Levels and Patient Outcomes: A Systematic Review of Longitudinal Studies, International Journal of Nursing Studies. (2022) 134, 10.1016/j.ijnurstu.2022.104311. 35780608

[61] Khan N. , Jackson D. , Stayt L. , and Walthall H. , Factors Influencing Nurses’ Intentions to Leave Adult Critical Care Settings, Nursing in Critical Care. (2019) 24, no. 1, 24–32, 10.1111/nicc.12348, 2-s2.0-85045113511. 29635820

[62] Goldman J. , Kitto S. , and Reeves S. , Examining the Implementation of Collaborative Competencies in a Critical Care Setting: Key Challenges for Enacting Competency-based Education, Journal of Interprofessional Care. (2018) 32, no. 4, 407–415, 10.1080/13561820.2017.1401987, 2-s2.0-85034665482. 29161170

[63] Graham F. , Eaton E. , Jeffrey C. , Secher-Jorgensen H. , and Henderson A. , Specialling” and “Sitters”: What Does Communication Between Registered Nurses and Unregulated Workers Reveal About Care?, Collegian. (2021) 28, no. 5, 482–488, 10.1016/j.colegn.2020.12.004.

[64] Sung T. C. and Hsu H. C. , Improving Critical Care Teamwork: Simulation-Based Interprofessional Training for Enhanced Communication and Safety, Journal of Multidisciplinary Healthcare. (2025) 18, 355–367, 10.2147/jmdh.s500890. 39872869 PMC11769723

