# Supplementary material for: Integration of Care Assistants Into Intensive Care Nursing Teams: A Multimethod Study
Source: Nurs Res Pract. 2026 Apr 30;2026:8153123. doi: 10.1155/nrp/8153123 (PMC13131058; doi:10.1155/nrp/8153123)
Supplement: Supplementary file 1 — Supporting Information 1 Supporting File 1. Complete questionnaires 1, 2 and 3. [file NRP-2026-8153123-s002.docx]

## Supplemental File 1

### Introduction

*Dear intensive care nurse,*

*A pilot project introducing care assistants into the ICU team will soon start in your department. As an ICU nurse, you will work closely with this new professional group in providing clinical care to patients and their families in the ICU.*

*To effectively evaluate the project, we ask for your opinions and experiences at various time points, starting with this initial survey. This will help us to understand ICU nurses’ expectations before the project begins and assess your current job satisfaction.*

*Completing the survey will take approximately 5 to 10 minutes. All responses are processed anonymously, with only the researchers having access to the data. Results will be presented as average scores at group level and will not be traceable to individual responses. More details can be found in the Participant Information Sheet.*

*The questionnaire includes several questions and statements. Please follow the instructions provided.*

*Select the circle next to your chosen answer. If you make a mistake, you can cross out the incorrect answer and select a different option.*

|  | Strongly disagree | Disagree | Agree | Strongly agree |
| --- | --- | --- | --- | --- |
| I generally view the developments regarding the integration of care assistants within the ICU work environment positively. |  |  |  |  |

*For substantive questions about the pilot project, please contact your team manager. For questions regarding this research, you can reach out to the research team.*

*Thank you in advance for completing the survey.*

### Questionnaire 1 – Evaluation pilot care assistants on the intensive care unit

The following questions pertain to your general information.

1. What is your gender?

- Male
- Female
- Other, namely ________
- Prefer not to say

1. What is your age?

- 20-30 years
- 31-40 years
- 41-50 years
- 51-60 years
- Older than 61 years
- Prefer not to say

1. What is the level of education regarding your current work position?

- Nurse EQF level 4 currently in training for ICU specialisation
- Nurse EQF level 4 with ICU specialisation
- Nurse EQF level 6 currently in training for ICU specialisation
- Nurse EQF level 6 with ICU specialisation
- Other, namely ________

1. How many years of work experience do you have in the ICU?

- Less than 1 year
- 1 – 5 years
- 6 – 10 years
- 11 – 20 years
- More than 21 years

1. Which department do you work in most frequently?

- Cardiothoracic adult ICU
- Medical/surgical adult ICU

The following questions pertain to the pilot project involving care assistants within the nursing team on the ICU. Please indicate the extent to which you agree with the statements.

|  | Strongly disagree | Disagree | Agree | Strongly agree |
| --- | --- | --- | --- | --- |
| 1. I generally view the developments regarding the integration of care assistants within the ICU work environment positively |  |  |  |  |
| *Would you like to elaborate on your response to the previous question?* |  | | | |
| 2. I think that expanding the nursing team with professionals from different educational backgrounds is valuable. |  |  |  |  |
| 3. I think it is challenging to maintain the quality of care with care assistants in the ICU work environment. |  |  |  |  |
| *Would you like to elaborate on your response to the previous question?* |  | | | |
| 4. Various care tasks in the ICU are well-suited for role differentiation. |  |  |  |  |
| 5. I am pleased to have care assistants in the ICU environment to perform essential basic care. |  |  |  |  |
| 6. I think it is challenging to maintain patient safety with care assistants in the ICU work environment. |  |  |  |  |
| *Would you like to elaborate on your response to the previous question?* |  | | | |
| 7. If care assistants are introduced in ICU care, there must be clarity about responsibilities and authorities. |  |  |  |  |
| 8. I have confidence in collaborating with care assistants in the ICU work environment. |  |  |  |  |
| *Would you like to elaborate on your response to the previous question?* |  | | | |

How satisfied are you with your work as an ICU nurse?

- Very dissatisfied
- Dissatisfied
- Satisfied
- Very satisfied

If possible, would you leave the hospital within the next year due to job dissatisfaction?

- Yes
- No

If you answered 'Yes,' what type of work would you be looking for?

- Nursing work in another hospital
- Nursing work, but not in a hospital
- A job outside the healthcare sector
- Other
- No work

Would you recommend the hospital to a fellow nurse as a good place to work?

- Definitely not
- Probably not
- Probably yes
- Definitely yes

What rating would you give to the quality of nursing care on your ward?

1 means you consider the quality poor, and a 10 stands for excellent.

| Quality of nursing care on your ward | | | | | | | | | | | |
| --- | --- | --- | --- | --- | --- | --- | --- | --- | --- | --- | --- |
| 1 | 2 | 3 | 4 | 5 | 6 | 7 | 8 | 9 | 10 | I don’t know |  |
|  |  |  |  |  |  |  |  |  |  |  |  |

**This is the end of the survey, thank you for completing it.**

### Questionnaire 2 – Evaluation pilot care assistants on the intensive care unit

The following questions pertain to your general information.

1. What is your gender?

- Male
- Female
- Other, namely ________
- Prefer not to say

1. What is your age?

- 20-30 years
- 31-40 years
- 41-50 years
- 51-60 years
- Older than 61 years
- Prefer not to say

1. Welke opleiding heb je gevolgd met betrekking tot je huidige functie?

- Nurse EQF level 4 currently in training for ICU specialisation
- Nurse EQF level 4 with ICU specialisation
- Nurse EQF level 6 currently in training for ICU specialisation
- Nurse EQF level 6 with ICU specialisation
- Other, namely ________

1. How many years of experience do you have in the ICU?

- Less than 1 year
- 1 – 5 years
- 6 – 10 years
- 11 – 20 years
- More than 21 years

1. Which department do you work in most frequently?

- Cardiothoracic adult ICU
- Medical/surgical adult ICU

**Part 1 – Evaluation pilot**

The following questions pertain to the pilot project with care assistants in the ICU nursing team. Please indicate the extent to which you agree with the statements.

|  | Strongly disagree | Disagree | Agree | Strongly  agree |
| --- | --- | --- | --- | --- |
| 1. I was well informed in advance about the pilot project with care assistants in my nursing team. |  |  |  |  |
| 2. ICU nurses had sufficient input in the preparation of the pilot. |  |  |  |  |
| 3. ICU nurses had sufficient input in the execution of the pilot. |  |  |  |  |
| 4. I think working with care assistants in the team is a positive development. |  |  |  |  |
| 5. I have the impression that care assistants know what their tasks are. |  |  |  |  |
| 6. I have more time to perform the complex care for ICU patients thanks to the involvement of care assistants. |  |  |  |  |
| 7. I generally collaborate well with care assistants. |  |  |  |  |
| 8. I experience increased workload since working with care assistants. |  |  |  |  |
| 9. I feel supported by my team manager. |  |  |  |  |
| 10. My team manager could do more to ensure a smooth integration of care assistants into the team. |  |  |  |  |
| *Would you like to elaborate on your response to the previous question?* |  | | | |
| 11. I am concerned that the quality of care is insufficiently safeguarded with the addition of care assistants to the nursing team. |  |  |  |  |
| *Would you like to elaborate on your response to the previous question?* |  | | | |

**Part 2 – Open questions**

1. What have been your experiences so far in collaborating with care assistants?

|  |
| --- |

1. What adjustments do you think are necessary for the integration of care assistants into the permanent ICU team?
   Please explain in your answer what went well or less well.

|  |
| --- |

1. Do you think that the pilot project with care assistants in the ICU should be continued in a permanent structure?

- Yes
- No

*Would you like to elaborate on your response to the previous question?*

|  |
| --- |

**Part 3 – Role differentiation and your working conditions**

This part of the questionnaire is about staffing and your working conditions. It is possible that a question may be less relevant or not applicable to your work as an ICU nurse. In that case, choose the answer that fits best your situation.

The first questions are about your work.

1. To what extent do you feel the following elements are present in your work?

| Aspects in ICU nursing work environment | | | | | |
| --- | --- | --- | --- | --- | --- |
|  | Extent of presence | | | | |
|  | Strongly disagree | Disagree | Agree | Strongly  agree | Not applicable to my situation |
| 1. Support services allow me to spend adequate time with my patients. |  |  |  |  |  |
| 2. Physicians and nurses have good working relationships. |  |  |  |  |  |
| 3. A supervisory staff that is supportive of the nurses. |  |  |  |  |  |
| 4. Active staff development or continuing education programs for nurses. |  |  |  |  |  |
| 5. Career development / clinical ladder opportunity |  |  |  |  |  |
| 6. Opportunity for staff nurses to participate in policy decisions. |  |  |  |  |  |
| 7. Doctors value nurses’ observations and judgments |  |  |  |  |  |
| 8. Enough time and opportunity to discuss patient care problems with other nurses. |  |  |  |  |  |
| 9. Enough registered nurses to provide quality patient care. |  |  |  |  |  |
| 10. A nurse manager who is a good manager and leader. |  |  |  |  |  |

2. How satisfied are you with your work as an ICU nurse?

- Very dissatisfied
- Dissatisfied
- Satisfied
- Very satisfied

3. How would you generally rate the work environment of your current job (such as adequate provision of resources, relationships with colleagues, and support from supervisors)?

- Poor
- Fair
- Good
- Excellent

4. How satisfied are you with the following aspects of your work?

|  | Strongly disagree | Disagree | Agree | Strongly  agree | Not applicable to my situation |
| --- | --- | --- | --- | --- | --- |
| 1. Vacation days |  |  |  |  |  |
| 2. Autonomy in work |  |  |  |  |  |
| 3. Study leave |  |  |  |  |  |
| 4. Professional status |  |  |  |  |  |
| 5. Salary |  |  |  |  |  |
| 6. Sick leave |  |  |  |  |  |
| 7. Career development opportunities |  |  |  |  |  |
| 8. Flexible work schedules |  |  |  |  |  |
| 9. Training opportunities |  |  |  |  |  |

5. If possible, would you leave the hospital within the next year due to dissatisfaction with your work?

- Yes
- No

*If you answered 'no' to question 5, proceed to question 7.*

6. If you answered 'Yes', what type of work would you be looking for?

- Nursing work in another hospital
- Nursing work, but not in a hospital
- A job outside the healthcare sector
- Other
- No work

7. How easy do you think it would be for you to find an acceptable job as an (ICU) nurse?

- Very difficult
- Fairly difficult
- Fairly easy
- Very easy

8. Would you recommend the hospital to a fellow nurse as a good place to work?

- Definitely not
- Probably not
- Probably yes
- Definitely yes

9. Would you recommend the hospital to your friends or family if they needed care?

- Definitely not
- Probably not
- Probably yes
- Definitely yes

10. What rating would you give to the quality of nursing care on your ward?

*A 1 means you consider the quality poor, and a 10 stands for excellent.*

| Quality of nursing care on your department | | | | | | | | | | | |
| --- | --- | --- | --- | --- | --- | --- | --- | --- | --- | --- | --- |
| 1 | 2 | 3 | 4 | 5 | 6 | 7 | 8 | 9 | 10 | I don’t know |  |
|  |  |  |  |  |  |  |  |  |  |  |  |

**The next questions pertain to your most recent shift.**

11. Which time period below best describes your most recent shift?

- Day
- Afternoon/Evening
- Night

12*.* Please write in the box the number of hours you worked during your most recent shift. *Please respond in whole hours (only the number of hours: for example, “8” and not “8 hours”)*.

|  |
| --- |

13. Did you work more than your contractual hours during your most recent shift?

- Yes
- No

14. How many patients were you directly responsible for during your most recent shift?

|  |
| --- |

15*.* Is the number of patients mentioned in the previous question typical for your workload? On average, the number of patients is:

- Less
- Same
- More

16. How would you describe your role in caring for most of the patients during your most recent shift? Please select the option that best fits.

- I provided most of the care myself
- I supervised care provided by others and also provided some care myself
- I provided limited care, such as wound care or administering medication, while most direct care was provided by others.
- I did not provide care, but focused on quality and organisational tasks.

17. How many patients were there in total on the ward during your most recent shift?

|  |
| --- |

18. Including yourself, how many nurses in total provided direct patient care during your most recent shift?

|  |
| --- |

19. Including yourself, how many nurses were present during your most recent shift? This is the number you provided in the previous question, including all nurses with other roles (such as team managers, clinical supervisors, or those in workgroups).

|  |
| --- |

20. How many other healthcare providers (care assistants and caregivers) in total provided direct patient care during your most recent shift?
Other healthcare providers refer to anyone other than certified MBO (EQF level 4) and HBO nurses (EQF level 6).

|  |
| --- |

21. How often did you perform the following tasks during your most recent shift?
*If the question does not apply to your situation, you may skip the question.*

|  | Never | Sometimes | Often | Always |
| --- | --- | --- | --- | --- |
| 1. Answering phones, clerical duties |  |  |  |  |
| 2. Obtaining supplies or equipment |  |  |  |  |
| 3. Filling in for non-nursing services not available on off-hours (outside normal working hours) |  |  |  |  |
| 4. Routine phlebotomy/blood draw for tests |  |  |  |  |
| 5. Performing non-nursing care |  |  |  |  |
| 6. Cleaning patient rooms and equipment |  |  |  |  |
| 7. Transporting of patients within hospital |  |  |  |  |
| 8. Arranging discharge referrals and transportation (including to long-term care facilities) |  |  |  |  |
| 9. Delivering and retrieving food trays |  |  |  |  |

22. Which of the following activities were necessary during your most recent shift but could not be completed due to lack of time? *Please check all that apply.*

- Planning care
- Oral hygiene
- Comfort/talking with patients and/or family
- Pain management
- Treatments and procedures (e.g. IV lines, catheters, changing wound dressings)
- Prepare patients and their families for discharge
- Skin care
- Adequate patient surveillance
- Educating patient and families
- Develop or update nursing care plans/care pathways
- Administer medications on time (according to HiX)
- Adequately document nursing care
- Frequent changing of patient position

23. How often have you acted in the following ways?

|  | Almost never | Sometimes | Occasionally | Usually | Almost always |
| --- | --- | --- | --- | --- | --- |
| 1. When I have concerns about my patient’s well-being, I ask critical questions about assignments and/or treatments. |  |  |  |  |  |
| 2. I support my clinical decisions with clinical reasoning and scientific insights. |  |  |  |  |  |
| 3. I reflect on my own and others' actions and strive to understand what went well and what did not. |  |  |  |  |  |
| 4. I negotiate with and support direct colleagues and colleagues from other disciplines to help patients achieve their personal goals. |  |  |  |  |  |
| 5. I feel engaged with patients when communicating with them about achieving personal goals. |  |  |  |  |  |
| 6. I conduct meaningful conversations with direct colleagues and colleagues from other disciplines to ensure we provide better, safer, and more person-centered care. |  |  |  |  |  |
| 7. I actively listen to the different opinions and perspectives of direct colleagues and colleagues from other disciplines. |  |  |  |  |  |
| 8. I build a therapeutic relationship with patients and their family members based on mutual trust and respect. |  |  |  |  |  |

24. To what extent do you recognise yourself in the following descriptions?

|  | Almost never | Sometimes | Occasionally | Usually | Almost always |
| --- | --- | --- | --- | --- | --- |
| 1. I develop collaborative relationships with my direct colleagues and colleagues from other disciplines, such as caregivers. |  |  |  |  |  |
| 2. I fulfil my promises and obligations to patients. |  |  |  |  |  |
| 3. I ensure that we work towards achievable goals with measurable objectives to achieve clinical outcomes for patients. |  |  |  |  |  |
| 4. I provide person-centered care. |  |  |  |  |  |
| 5. I publicly compliment direct colleagues and colleagues from other disciplines who are role models for professionalism. |  |  |  |  |  |
| 6. I give positive feedback to direct colleagues and colleagues from other disciplines who contribute to the well-being of patients and their families through their actions. |  |  |  |  |  |
| 7. I always find ways to show appreciation for the achievements of direct colleagues and colleagues from other disciplines. |  |  |  |  |  |

Indicate whether you agree or disagree with the two statements below:

25. “I consider myself a clinical leader in my work”

- Agree
- Disagree

26. “I exhibit leadership behavior in my daily work.”

- Agree
- Disagree

*27.* To what extent do you find the following elements present in your work?

|  | Extent of presence | | | | |
| --- | --- | --- | --- | --- | --- |
|  | Strongly disagree | Disagree | Agree | Strongly agree | Not applicable to my situation |
| 1. Much teamwork between nurses and doctors |  |  |  |  |  |
| 2. Opportunities for career development/Opportunities for advancement. |  |  |  |  |  |
| 3. Enough staff to get the work done. |  |  |  |  |  |
| 4. Praise and recognition for a job well done. |  |  |  |  |  |
| 5. Working with nurses who are clinically competent. |  |  |  |  |  |
| 6. High standards of nursing care are expected by the administration. |  |  |  |  |  |
| 7. Doctors recognise the contribution of nurses to patient care/ The contributions that nurses make to patient care are publicly acknowledged. |  |  |  |  |  |
| 8. A chief nursing executive is equal in power and authority to other top-level hospital. |  |  |  |  |  |
| 9. Doctors respect nurses as equal professionals |  |  |  |  |  |
| 10. The environment in which patient care is provided is characterised by a clear nursing vision/ Primary nursing as the nursing delivery system |  |  |  |  |  |
| 11. A chief nursing officer is highly visible and accessible to staff. |  |  |  |  |  |

*28.* To what extent do you agree with the following statements?

|  | Extent of presence | | | | |
| --- | --- | --- | --- | --- | --- |
|  | Strongly disagree | Disagree | Agree | Strongly agree | Not applicable to my situation |
| 1. An administration that listens and responds to employee concerns. |  |  |  |  |  |
| 2. A nurse manager backs up the nursing staff in decision making, even if the conflict is with a physician. |  |  |  |  |  |
| 3. (Staff) nurses are involved in the internal governance of the hospital (e.g., practice and policy committees). |  |  |  |  |  |
| 4. Collaboration (joint practice) between nurses and physicians |  |  |  |  |  |
| 5. Nursing care is based on a nursing rather than a medical model. |  |  |  |  |  |
| 6. Written, up-to-date nursing care plans for all patients. |  |  |  |  |  |
| 7. A good orientation program for newly employed nurses |  |  |  |  |  |
| 8. (Staff) Nurses have the opportunity to be part of hospital and nursing committees. |  |  |  |  |  |
| 9. Patient assignments foster continuity of care (i.e, the same nurse cares for the patient from one day to the next). |  |  |  |  |  |
| 10. An active quality-assurance program. |  |  |  |  |  |
| 11. Doctors have a high regard for nurses. |  |  |  |  |  |

Do you have any comments or suggestions?

|  |
| --- |

**This is the end of the questionnaire, thank you for completing it.**

### Questionnaire 3 – Evaluation pilot care assistants on the intensive care unit

The following questions pertain to your general information.

1. What is your gender?

- Male
- Female
- Other, namely ________
- Prefer not to say

1. What is your age?

- 20-30 years
- 31-40 years
- 41-50 years
- 51-60 years
- Older than 61 years
- Prefer not to say

1. How many years of experience do you have in the ICU?

- Less than 1 month
- 1 – 2 months
- 3 months
- More than 3 months

1. Which department do you work in most frequently?

- Cardiothoracic adult ICU
- Medical/surgical adult ICU

The following questions pertain to your work in the ICU nursing team. Please indicate the extent to which you agree with the statements.

|  | Strongly disagree | Disagree | Agree | Strongly  agree |
| --- | --- | --- | --- | --- |
| The information I received about working in the ICU was sufficient for me. |  |  |  |  |
| I felt welcomed in the ICU. |  |  |  |  |
| I was properly trained in ICU care. |  |  |  |  |
| I know which tasks I need to perform. |  |  |  |  |
| I feel comfortable performing the tasks assigned to me. |  |  |  |  |
| *Would you like to elaborate on your response to the previous question?* |  | | | |
| Collaboration with ICU nurses is generally pleasant. |  |  |  |  |
| I find working in an ICU usually a fun challenge. |  |  |  |  |
| I find working in an ICU (too) stressful. |  |  |  |  |
| I feel that I am overly directed by ICU nurses in the tasks I perform. |  |  |  |  |
| I feel uncertain in collaborating with ICU nurses. |  |  |  |  |
| I know when to alert an ICU nurse. |  |  |  |  |
| The work in the ICU aligns with my knowledge and skills. |  |  |  |  |
| I find it easy to support families in the ICU, such as offering a diary or a comforting conversation. |  |  |  |  |
| I would recommend working in an ICU to fellow care assistants. |  |  |  |  |
| I feel valued as a colleague in the ICU team. |  |  |  |  |
| I experience high work pressure. |  |  |  |  |
| I feel supported by my supervisor. |  |  |  |  |
| My supervisor could do more to facilitate smoother team integration. |  |  |  |  |
| *Would you like to elaborate on your response to the previous question?* |  | | | |
| I feel like I am part of the care for ICU patients / I contribute (professionally) to the care of ICU patients. |  |  |  |  |
| *Would you like to elaborate on your response to the previous question?* |  | | | |

How satisfied are you with your work as a care assistant?

- Very dissatisfied
- Dissatisfied
- Satisfied
- Very satisfied

If possible, would you leave the hospital within the next year due to job dissatisfaction?

- Yes
- No

If you answered 'Yes,' what type of work would you be looking for?

- Care assistant work in another hospital
- Care assistant work, but not in a hospital
- A job outside the healthcare sector
- Other
- No work

Would you recommend the hospital to a fellow care assistant as a good place to work?

- Definitely not
- Probably not
- Probably yes
- Definitely yes

What rating would you give to the quality of nursing care on your ward?

1 means you consider the quality poor, and a 10 stands for excellent.

| Quality of nursing care on your ward | | | | | | | | | | | |
| --- | --- | --- | --- | --- | --- | --- | --- | --- | --- | --- | --- |
| 1 | 2 | 3 | 4 | 5 | 6 | 7 | 8 | 9 | 10 | I don’t know |  |
|  |  |  |  |  |  |  |  |  |  |  |  |

**Open questions**

What have been your experiences so far in collaborating with ICU nurses?

|  |
| --- |

Do you have any additional ideas on how the tasks could better align with your knowledge and skills?

|  |
| --- |

**This is the end of the survey, thank you for completing it.**
