# Supplementary material for: Integration of Care Assistants Into Intensive Care Nursing Teams: A Multimethod Study
Source: Nurs Res Pract. 2026 Apr 30;2026:8153123. doi: 10.1155/nrp/8153123 (PMC13131058; doi:10.1155/nrp/8153123)
Supplement: Supplementary file 2 — Supporting Information 2 Supporting File 2. Narratives and expectations of ICU nurses. [file NRP-2026-8153123-s001.docx]

## Supplemental File 2

### Narratives of comments in questionnaire 1 – expectations of integrated teams

Some respondents believed that care assistants could positively contribute to both patient safety and care quality. One expressed that having more staff could improve monitoring: *“More people on the unit means more eyes on the patient,”* while another added, *“As long as tasks, responsibilities, and competencies are clear to all colleagues, the quality of care might actually improve.”* This conditional optimism was echoed by another respondent, who stated that “care assistants can definitely be of value to the team if we train them properly and set clear agreements.” However, others stressed that care assistants might struggle with complex tasks due to a lack of experience: *“It seems much harder for them to see the full picture due to lack of knowledge,”* and *“The expertise of a nurse is greater than that of a care assistant. I'm concerned that the nurse's oversight will diminish.”* Some respondents indicated that this made it difficult to form a clear opinion about the initiative, questioning whether the scope of tasks assigned to care assistants was realistic: *“I find it difficult to form an opinion, as their scope of tasks seems broad, and I question whether it’s realistic.”* Differences in training were also highlighted, with one respondent noting: *“ICU nurses need 18 months of training after their nursing degree, and care assistants only get one week?”*

Several respondents raised concerns about accountability, noting: *“Care assistants are responsible for their actions, but who holds ultimate responsibility for the patient?"* Additionally, there was scepticism about care assistants being adequately prepared for the demands of ICU care: *“I am afraid that care assistants will take over more basic care, and I'll have more ICU patients to manage. That worries me.”* Many respondents also worried about maintaining patient safety in the ICU. One respondent stated, *“Patient safety for vulnerable ICU patients is potentially at risk if a care assistant, rather than an ICU nurse, takes over as the second caregiver.”* Another echoed this doubts, remarking, *“They are not used to delivering this complex care, and I will need to stay alert as an ICU nurse to ensure the patient isn't put at risk.”*

Several respondents expressed uncertainties that integrating care assistants in the team might fragment care delivery, leading to reduced oversight and potential drops in care quality. In some cases, respondents perceived the initiative as a temporary or suboptimal solution to staffing shortages rather than a sustainable change, stating that “I think this is a step backward, not forward. It feels like a stopgap solution to fill staffing shortages.” One stated, *“I'm worried that things will become fragmented, and I won't be able to observe everything myself anymore.”* Others emphasized the risk of losing important patient information: *“If care assistants take on tasks, important observations might be missed, and patient care could suffer.”*

Regarding collaboration, many respondents expressed optimism and trust in working with care assistants. There was a general belief that, with proper integration and training, the collaboration could be successful. One respondent noted, *“I have full confidence in a good collaboration.”* Others acknowledged that, while it may take time for the team to adjust, trust would develop gradually: *“Collaboration will happen, but it will take time for everyone to get used to it and gain trust in delegating tasks.”* Several respondents highlighted the potential for workload relief, provided care assistants were well-trained: *“I trust that we can achieve significant workload relief if we train the care assistants well.”*

Some respondents expressed cautious optimism, recognizing that the outcome would depend on team dynamics and clear task distribution. As one respondent noted, *“The first three months will tell us a lot about how this will go. It also depends on how well the team welcomes the care assistants and integrates them on the floor.”* Another expressed concern about the level of responsibility given to care assistants: *“I am quite sceptical, especially regarding quality and responsibility.”* Others emphasized the importance of clearly defined roles: *“Care assistants can be a good addition, provided the tasks are clearly divided.”* In addition, some respondents expressed concern about the potential impact of the role on care assistants themselves. There was apprehension that the position might primarily involve undesirable tasks and offer limited professional value, with one respondent remarking, “I fear care assistants might end up doing only unpleasant tasks, which adds no real value for them or us.”

### Narratives of comments in questionnaire 2 – experiences with integrated teams

A few respondents expressed dissatisfaction, feeling that their managers could do more to support the integration of care assistants. One remarked, *"I get the feeling that this needs to succeed, whether we agree with it or not. They call it a pilot, but it’s not."* Additionally, some respondents raised concerns about a lack of visible support from management, noting, *"There is no noticeable connection between the managers and the team,"* and *"I haven’t noticed much interaction between the managers and the care assistant*s." In contrast, other participants felt that managerial involvement was sufficient, stating, *“I think my manager is doing enough in this.”*

After three months of integration, some participants expressed confidence in the potential of care assistants, highlighting their contributions in a supportive role. One respondent stated, *"I'm not worried that care quality is at risk; they are a good addition to our team."* Another noted, *"If they are well-trained, care assistants can eventually add value, but it will take time and energy to develop them."* At the same time, others described a more conditional form of collaboration, particularly during high-pressure situations: *“Currently, we’ve found a reasonable way of working. We handle patient care, and care assistants help, but their contributions aren't significant during high-pressure shifts.”* Some respondents also suggested that, with proper delegation, their presence could be beneficial: *"If care assistants handle low-complexity tasks, ICU nurses could focus more on high-complexity care."* However, many respondents expressed significant concerns about the integration of care assistants, particularly regarding risks to patient safety and the quality of care. One participant pointed out, *"The level difference is noticeable; I spend more time checking their work, which makes my job longer instead of helping."* Another added, *"They aren't trained for this category of patients, and I’m concerned that care quality won’t be maintained."* Others emphasized similar concerns, stating that *"Care assistants aren't qualified to work in an ICU; they lack the necessary knowledge, and if something happens, they can't respond appropriately."* Others pointed out that their involvement could even increase the workload: *"I feel like I'm constantly inventing tasks for them to do, rather than being relieved."*

### Suggested adaptations for integrating care assistants into the ICU team

Respondents offered a range of suggestions for adapting the integration of care assistants into the ICU team. Several respondents (n=8) suggested further training and education as critical to improving the role of care assistants, with recommendations for specific training in ICU-related skills, such as medication management, the ‘ABCDE approach’, and reanimation techniques. Others (n=6) emphasized the need for a clearer task description and division of responsibilities, with some calling for regular evaluations to clarify which tasks care assistants are permitted to perform. Several participants (n=4) felt that assigning care assistants to lower-complexity tasks or providing more direct supervision would enhance their role in the ICU.

A few respondents (n=5) advocated for a structured mentorship to help care assistants transition smoothly into the ICU environment. Others (n=6) questioned the appropriateness of having care assistants on a high-complexity ICU, suggesting that they may be better suited for work on a general ward or that their current level of involvement compromises patient safety. Some also mentioned (n=3) that fostering a positive team culture and involving care assistants more actively in team activities would improve collaboration.
